# Analysis of *Corynebacterium silvaticum* genomes from Portugal reveals a single cluster and a clade suggested to produce diphtheria toxin

**DOI:** 10.7717/peerj.14895

**Published:** 2023-03-09

**Authors:** Marcus Vinicius Canario Viana, José Henrique Galdino, Rodrigo Profeta, Manuela Oliveira, Luís Tavares, Siomar de Castro Soares, Paulo Carneiro, Alice Rebecca Wattam, Vasco Azevedo

**Affiliations:** 1 Department of Genetics, Ecology and Evolution, Institute of Biological Sciences, Federal University of Minas Gerais, Belo Horizonte, Minas Gerais, Brazil; 2 Department of Biological Sciences, State University of Southwest of Bahia, Jequié, Bahia, Brazil; 3 Centre for Interdisciplinary Research in Animal Health, Faculty of Veterinary Medicine, University of Lisbon, Lisbon, Portugal; 4 Department of Immunology, Microbiology and Parasitology, Institute of Biological Sciences and Natural Sciences, Federal University of Triângulo Mineiro, Uberaba, Minas Gerais, Brazil; 5 Biocomplexity Institute, University of Virginia, Charlottesville, VA, United States of America

**Keywords:** Corynebacterium silvaticum, Pathogen, Diphtheria

## Abstract

**Background:**

*Corynebacterium silvaticum* is a pathogenic, gram-positive bacterial species that causes caseous lymphadenitis in wild boars, domestic pigs and roe deer in Western Europe. It can affect animal production and cause zoonosis. Genome analysis has suggested that one strain from Portugal and one from Austria could probably produce the diphtheria toxin (DT), which inhibits protein synthesis and can cause death.

**Methods:**

To further investigate the species genetic diversity and probable production of DT by Portuguese strains, eight isolates from this country were sequenced and compared to 38 public ones.

**Results:**

Strains from Portugal are monophyletic, nearly identical, form a unique cluster and have 27 out of 36 known *Corynebacterium* virulence or niche factors. All of them lack a frameshift in the *tox* gene and were suggested to produce DT. A phylogenetic analysis shows that the species has diverged into two clades. Clade 1 is composed of strains that were suggested to have the ability to produce DT, represented by the monophyletic strains from Portugal and strain 05-13 from Austria. Clade 2 is composed of strains unable to produce DT due to a frameshifted *tox* gene. The second clade is represented by strains from Austria, Germany and Switzerland. Ten genome clusters were detected, in which strains from Germany are the most diverse. Strains from Portugal belong to an exclusive cluster. The pangenome has 2,961 proteins and is nearly closed (*α* = 0.968). Exclusive genes shared by clusters 1 and 2, and Portuguese strains are probably not related to disease manifestation as they share the same host but could play a role in their extra-host environmental adaptation. These results show the potential of the species to cause zoonosis, possibly diphtheria. The identified clusters, exclusively shaded genes, and exclusive STs identified in Portugal could be applied in the identification and epidemiology of the species.

## Introduction

*Corynebacterium silvaticum* is a species of recently described gram-positive pathogenic bacteria (Dangel et al., 2020) that has been isolated from wild board and roe deer in Germany (Dangel et al., 2020; Möller et al., 2020a), Austria (JABGCO01), Switzerland (JAEANX01), and from domestic pigs in Portugal (Oliveira et al., 2014; Viana et al., 2020). The infection manifests in a disease similar to caseous lymphadenitis (CL) (Dangel et al., 2020), which is caused by *C. pseudotuberculosis* in goats and sheep (Dorella et al., 2006). Prior to an analysis and designation of a new species, strains from this species were identified as *C. pseudotuberculosis* or *C. ulcerans* (Oliveira et al., 2014; Dangel et al., 2019; Rau et al., 2019).

*C. silvaticum* is part of a group of six phylogenetically related pathogenic species that include *C. diphtheriae*, *C. belfantii*, *C. rouxii*, *C. ulcerans*, and *C. pseudotuberculosis*, which can produce diphtheria toxin (DT) when lysogenized by *tox*^+^ corynephages (Bernard & Funke, 2015; Dangel et al., 2020; Badell et al., 2020). This toxin causes cell death by inactivating protein synthesis (Murphy, 2011). One of the characteristics of *C. silvaticum* is that it is non-toxigenic, yet it has the *tox* gene (NTTB) (Dangel et al., 2020), caused by a frameshift in *tox* (Viana et al., 2020). The Portuguese strain PO100/5 and Austrian strain 05-13 do not have the characteristic frameshift in the *tox* gene caused by the insertion of two guanines (Möller et al., 2020a; Viana et al., 2020), suggesting that those strains are producers of DT. The isolation from domestic pigs suggests a potential for zoonotic transmission (Viana et al., 2020). Besides production of DT, cytotoxicity in human epithelial cells has recently been demonstrated (Möller et al., 2021).

The possibility of zoonotic transmission and the impact it could have on animal production implicate *C. silvaticum* as a potential threat to human health. In this work, we investigated the genetic diversity of the species by sequencing eight genomes from Portugal, with the aim of identifying genomic features that could be used for its control.

## Materials & Methods

### Genome assembly and taxonomy

The eight *C. silvaticum* strains from domestic pigs in Portugal used in the analysis were isolated by Oliveira et al. (2014) (Data S1). At the time the strains were classified as *C. pseudotuberculosis*. The genomes were sequenced using Illumina HiSeq 2500 (Illumina, San Diego, CA, USA) with 2 × 150 bp paired-end libraries. The quality of the sequencing reads was assessed by FastQC v0.11.9 (Andrews, 2015). Each genome was assembled using both reference-based and *de novo* assemblies. For the reference-based assembly, we used as reference the first version of *C. silvaticum* PO100/5 genome (CP021417.1, BV-BRC 65058.108). The tool used for read mapping and extraction of consensus sequence was UGENE 39 (Okonechnikov et al., 2012), with the plugins Bowtie v2.4.2 (Langmead & Salzberg, 2012) and SAMtools v0.1.19 (Li et al., 2009). For the *de novo* assemblies, we performed three assemblies using SPAdes v3.15.3 (Bankevich et al., 2012), Unicycler v0.4.8 (Wick et al., 2017) and Edena v3.131028 (Hernandez et al., 2008). Before finishing the assembly, the taxonomy of the sample was determined using the type strain genome server (Meier-Kolthoff & Göker, 2019). Then, the best *de novo* assembly was determined by QUAST v5.1.0rc1 (Gurevich et al., 2013). This assembly was scaffolded using CONTIGuator v2 (Galardini et al., 2011) using CP021417.1 as reference. The beginning of the chromosome was moved to the *dnaA* gene using the script moveDNAA.py (https://github.com/dcbmariano/scripts/blob/master/moveDNAA.py). The gaps were automatically closed using the contigs of the other three assemblies using GFinisher v1.4 (Guizelini et al., 2016) with CP021417.1 as reference. The assembly completeness and contamination were evaluated using CheckM 2 (https://github.com/chklovski/CheckM2).

For comparison of *tox*^+^ prophages, we reassembled the public genomes of strains 05-13 ( SRR11485666) and KL0182^T^ (SRR7825394) (Data S1). The raw sequencing data was retrieved using fastq-dump from SRA Toolkit (https://github.com/ncbi/sratoolkit) and the assembly was performed with the method used in the strains from Portugal but replacing Edena’s assembly for the respective public assembly.

### Clustering, typing and annotation

Genome clusters were determined using PopPUNK v2.6.0 (Lees et al., 2019) and the network was visualized using Cytoscape v3.9.1 (Shannon et al., 2003). The sequence type (ST) of the strains was determined using MLST v 2.0.4 (Larsen et al., 2012). The genomes were annotated using the Rapid Annotation using Subsystems Technology (RASTtk) pipeline (Brettin et al., 2015), implemented in the Bacterial and Viral Bioinformatics Resource Center (BV-BRC) (Olson et al., 2022), and submitted to GenBank (Benson et al., 2012).

### Characterization of *C. silvaticum* genomes from Portugal

Plasmids, insertion sequences and prophages were predicted using PlasmidFinder v2.1 (Carattoli et al., 2014), ISEScan v1.7.2.3 (Xie & Tang, 2017) and PHASTER (Arndt et al., 2016), respectively. Genomics islands were predicted for strain PO100/5 using GIPSy v1.1.3 (Soares et al., 2016), with *C. glutamicum* ATCC13032 (CP025533.1) as a non-pathogenic reference. CRISPR-Cas systems were identified using CRISPRCasFinder (Couvin et al., 2018). Virulence factors were predicted with Abricate v1.0.1 https://github.com/tseemann/abricate), with minimum identity and coverage values of 60%. Virulence and niche factors were identified using BV-BRC’s Proteome Comparison Tool, using a list of 37 genes described for *C. silvaticum*, *C. ulcerans*, *C. pseudotuberculosis*, *C. diphtheriae*, *C. jeikeium* and *C. glutamicum* (Trost et al., 2010; Trost et al., 2011; Tauch & Burkovski, 2015; Reardon-Robinson et al., 2015; Weerasekera et al., 2019; Möller et al., 2020b). A circular map of PO100/5 was generated using BRIG v0.95 (Alikhan et al., 2011).

The *tox* gene sequence was compared across *Corynebacterium* species to look for the frameshift described in *C. silvaticum* (Dangel et al., 2020). The representatives for *C. silvaticum* included the KL0182^T^ and W25 strains from Germany, 5182 from Switzerland, 04-13 and 05-13 from Austria, and the eight strains from Portugal. Three other species were included in the comparison: *C. ulcerans* 0102 (AP012284.1), *C. pseudotuberculosis* 31 (CP003421.4) and *C. diphtheriae* NCTC13129 (NC_002935). The sequences were aligned using Jalview v2.11.1.4 (Waterhouse et al., 2009), with the MUSCLE algorithm (Edgar, 2004).

### Comparative genomics with other strains

Thirty-eight public *C. silvaticum* genomes were obtained from BV-BRC and GenBank (Data S1) for a total of 46 when the Portuguese genomes were included. For samples available as sequencing reads (Data S1), we used the assemblies performed by Viana et al. (2020). A phylogenomic tree of *C. silvaticum* was built using BV-BRC’s Phylogenetic Tree Building tool, using the nucleotide and amino acid sequences from 1,000 shared genes. *C. ulcerans* NCTC 7910^T^ was used as an external group. Average Nucleotide Identity (ANI) was calculated using FastANI v1.0 (Jain et al., 2018).

The distribution of orthologous gene groups across all genomes from all three species was estimated using OrthoFinder v2.5.4 (Emms & Kelly, 2019) and in-house scripts (Data S2). Here, the core genome is defined by orthogroups shared by all genomes, accessory genome is defined by orthogroups shared by more than one but not all genomes, and singletons are exclusive genes of a single genome. A pangenome is the entire repertoire of orthogroups found across all genomes. We used an in-house script to identify subsets of gene groups exclusively shared by (1) *C. silvaticum* strains from Portugal, (2) *C. silvaticum* strains from Portugal and strain 05-13 from Austria, (3) the remaining *C. silvaticum* strains.

The prophages of strains PO100/5, 05-13 and KL0182 were compared using tBLASTx v2.9.0+ or BLASTn v2.9.0+ (Camacho et al., 2009) and visualized using Artemis Comparison Tool (ACT) v18.1.0 (Carver et al., 2008; Carver et al., 2012). Possible misassemblies were investigated by mapping sequencing reads to the assembled genome or a reference using UGENE. The reassembled version and KL0182^T^ was also used for genomic island prediction using GISPy to represent strains out of Portugal.

## Results

### Characterization of *C. silvaticum* genomes from Portugal

**Figure 1:**
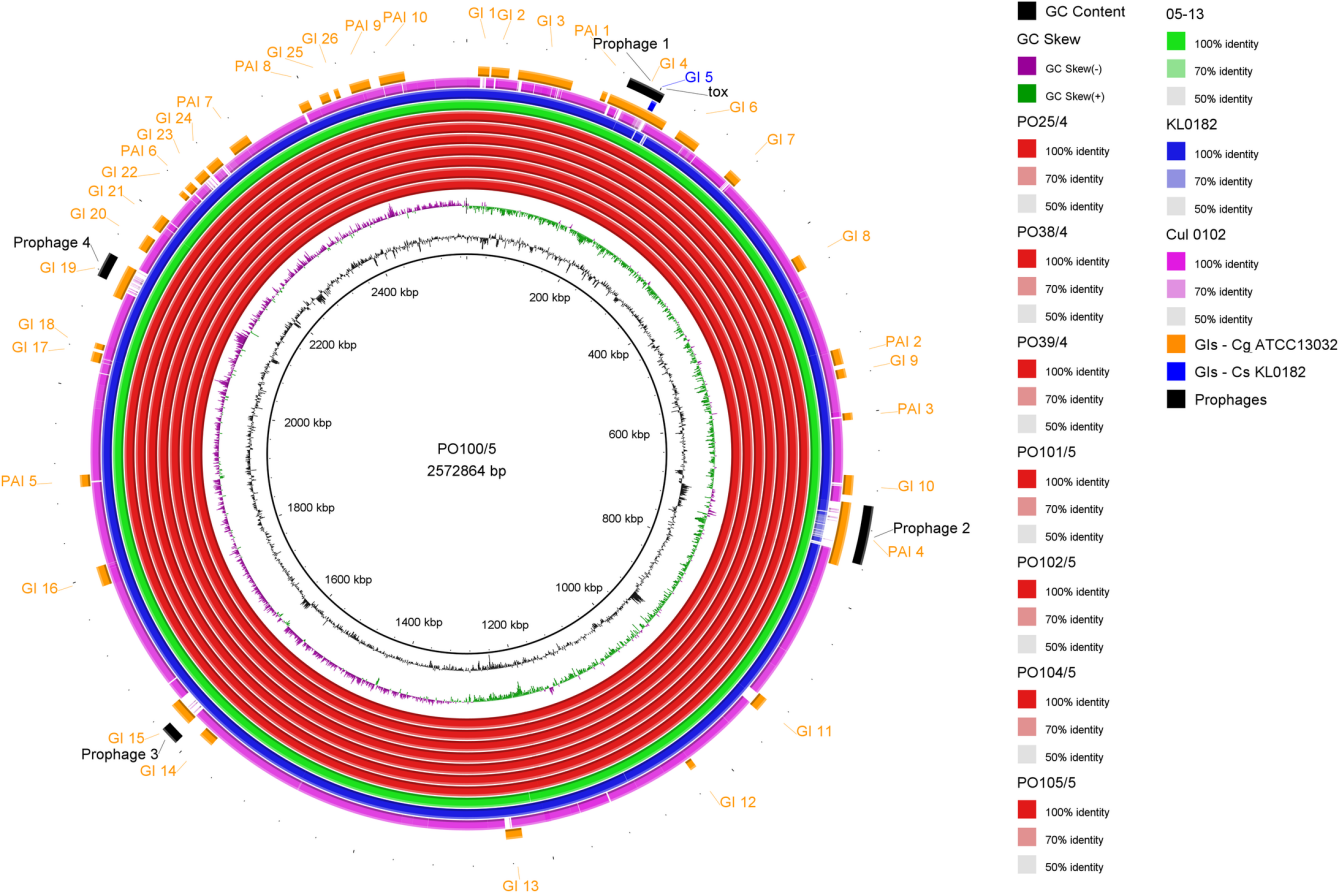
Circular map of *Corynebacterium silvaticum* PO100/5 genome generated using BRIG v0.95. Genomic islands and prophage detection were performed using GIPSy and PHASTER, respectively. Cul –*C. ulcerans*, GIs –genomic islands, Cg –*C. glutamicum*, Cs –*C. silvaticum*.

**Figure 2:**
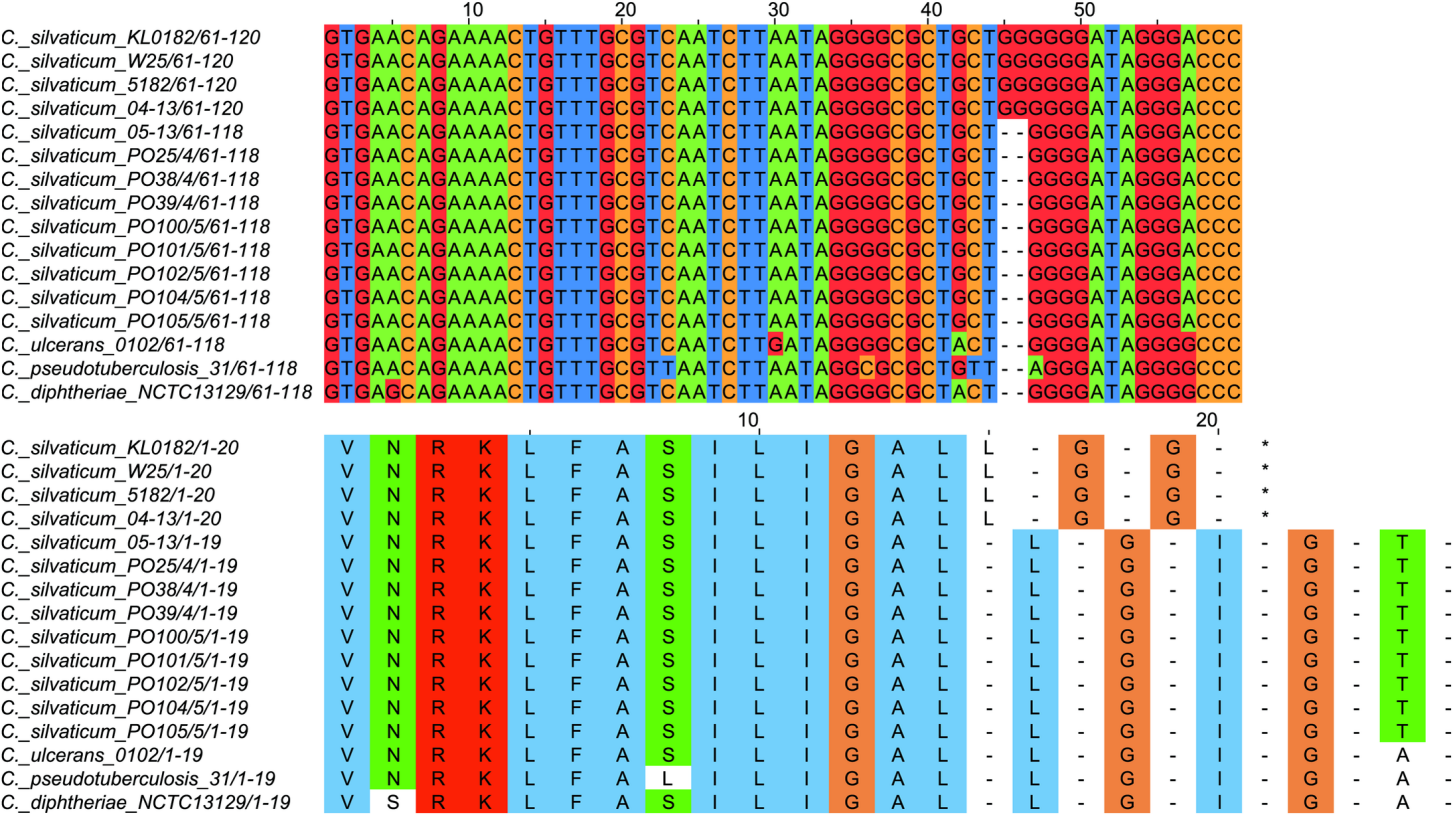
Alignment of the *tox* gene’s first 60 nucleotides and respective amino acids of strains from *Corynebacterium silvaticum*, *C. ulcerans*, *C. pseudotuberculosis* and *C. diphtheriae*. *C. silvaticum* strains from Portugal PO25/4, PO38/4, PO39/4, PO100/5, PO101/5, PO102/5, PO104/5 and PO105/5, and the strain 05-13 from Switzerland do not have the insertion of two guanines that led to a frameshift in other strains from this species. The frameshift results in a truncated protein with 17 amino acids. The alignment was performed using MUSCLE algorithm implemented in Jalview v2.11.1.4.

All strains from Portugal were identified as *C. silvaticum* (Data S3). The assemblies were estimated to be 99.9% complete with 0.19 or 0.2% contamination. No plasmids were detected. The genome sizes were ∼2.573 Mb, with 2,631 to 2,639 CDSs, 12 rRNA genes and 52 tRNA genes (Table 1). All genomes have three insertion sequences families (Table 1, Data S4), two complete and one to two incomplete prophages (Table 1, Data S5), and a Type I-E CRISPR-Cas system (Table 1, Data S6). The ANI values ranged from 99.9948 to 99.9998% (Data S7). The *tox*^+^ prophage was ∼38 Kb in all strains (Data S5). PO100/5 has 35 genomic islands in comparison to *C. glutamicum* ATCC13032 and one in comparison to KL0182^T^ (Fig. 1, Data S8). KL0182^T^ has 35 islands when compared to *C. glutamicum* (Data S8). Some islands are found in both PO100/5 and KL0182, and some are unique to each strain (Data S8). The new ST 795 was found in PO104/5, differing from the ST 578 by the new allele 65 of the gene *atpA* (Table 1, Data S9). Abricate identified virulence genes in four of the eight genomes (*tox*, *relA*, *ideR* and *ureB*) (Table 2, Data S10). The proteome comparison tool identified 28 out of 37 known *Corynebacterium* virulence or niche factors, although the pili genes *spaCDEF* were pseudogenized (Table 2, S11 File). All strains from Portugal and strain 05-13 from Austria have a *tox* gene that codes a DT with 560 amino acids with identical sequence (Data S12). The other strains have a frameshift that is caused by the insertion of two guanines in position 44 (Data S13). The frameshift results in a truncated protein of 17 amino acids (Fig. 2).

**Table 1:** Genome features of *Corynebacterium silvaticum* strains from Portugal.

**Strain**	**PO25/4**	**PO38/4**	**PO39/4**	**PO100/5**	**PO101/5**	**PO102/5**	**PO104/5**	**PO105/5**
Genbank accession	CP080461	CP081182	CP081179	CP021417.2	CP081180	CP080459	CP080460	CP081181
Completeness (%)	99.9	99.9	99.9	99.9	99.9	99.9	99.9	99.9
Contamination (%)	0.2	0.19	0.2	0.19	0.19	0.19	0.2	0.2
Size (bp)	2,572,825	2,572,864	2,572,860	2,572,864	2,572,991	2,572,936	2,572,895	2,572,843
Plasmid	–	–	–	–	–	–	–	–
CG content (%)	54.40	54.40	54.40	54.40	54.40	54.40	54.40	54.40
CDS	2,631	2,633	2,636	2,633	2,636	2,635	2,634	2,639
tRNA	52	52	52	52	52	52	52	52
rRNA	12	12	12	12	12	12	12	12
Repeat region	24	24	24	24	24	24	24	24
Sequence type	578	578	578	709	578	709	795	578
Insertion sequence	IS21, IS110 and IS256	IS21, IS110 and IS256	IS21, IS110 and IS256	IS21, IS110 and IS256	IS21, IS110 and IS256	IS21, IS110 and IS256	IS21, IS110 and IS256	IS21, IS110 and IS256
Prophages	2 questionable, 2 incomplete	2 questionable, 1 incomplete	2 questionable, 2 incomplete	2 questionable, 2 incomplete	2 questionable, 1 incomplete	2 questionable, 2 incomplete	2 questionable, 2 incomplete	2 questionable, 2 incomplete
CRISPR-Cas system	Type I-E	Type I-E	Type I-E	Type I-E	Type I-E	Type I-E	Type I-E	Type I-E

### Comparative genomics with other strains

**Figure 3:**
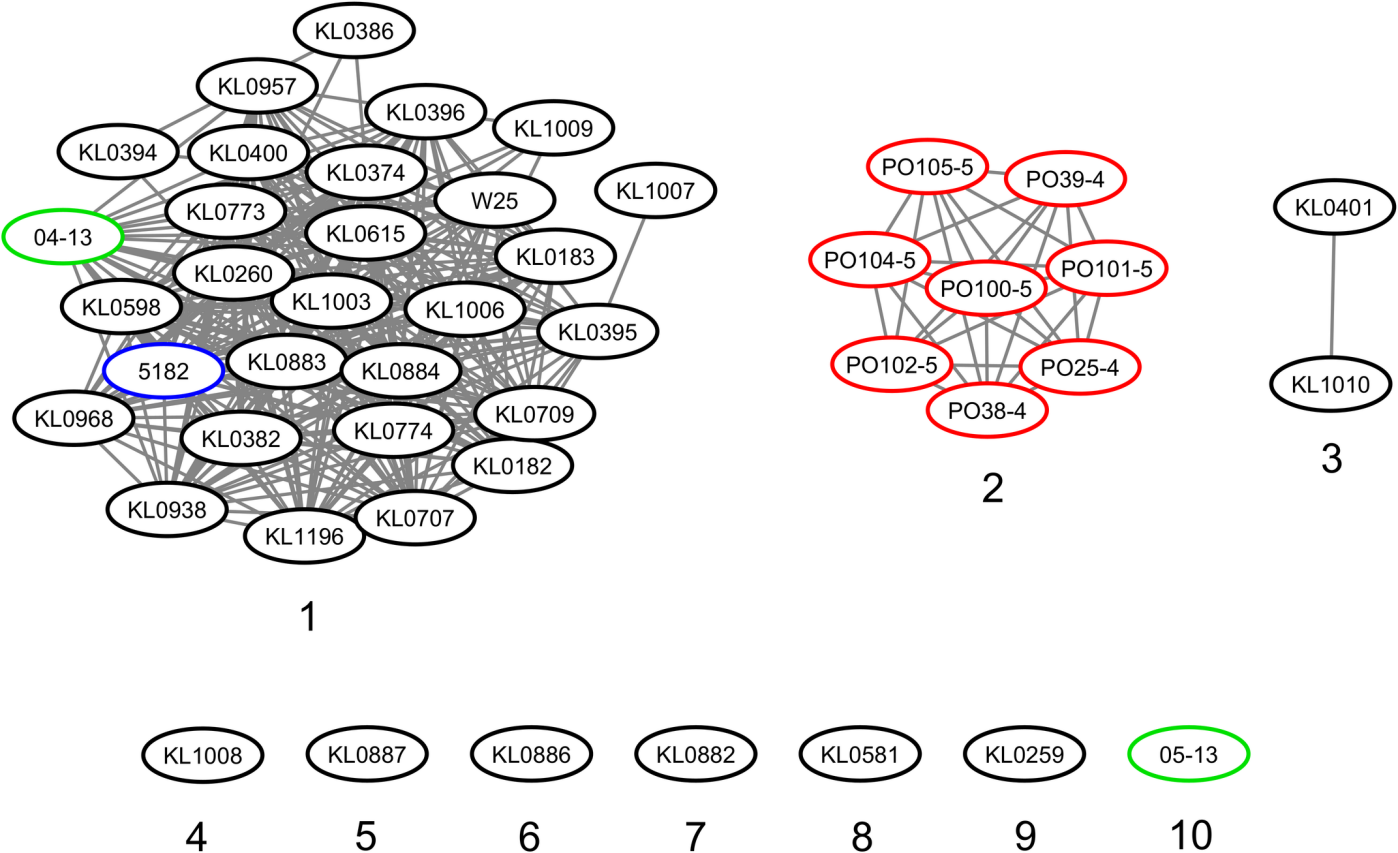
Clusters of *Corynebacterium silvaticum* genomes. Countries of sample isolation: Austria (green), Germany (black), Portugal (red), Switzerland (blue).

**Figure 4:**
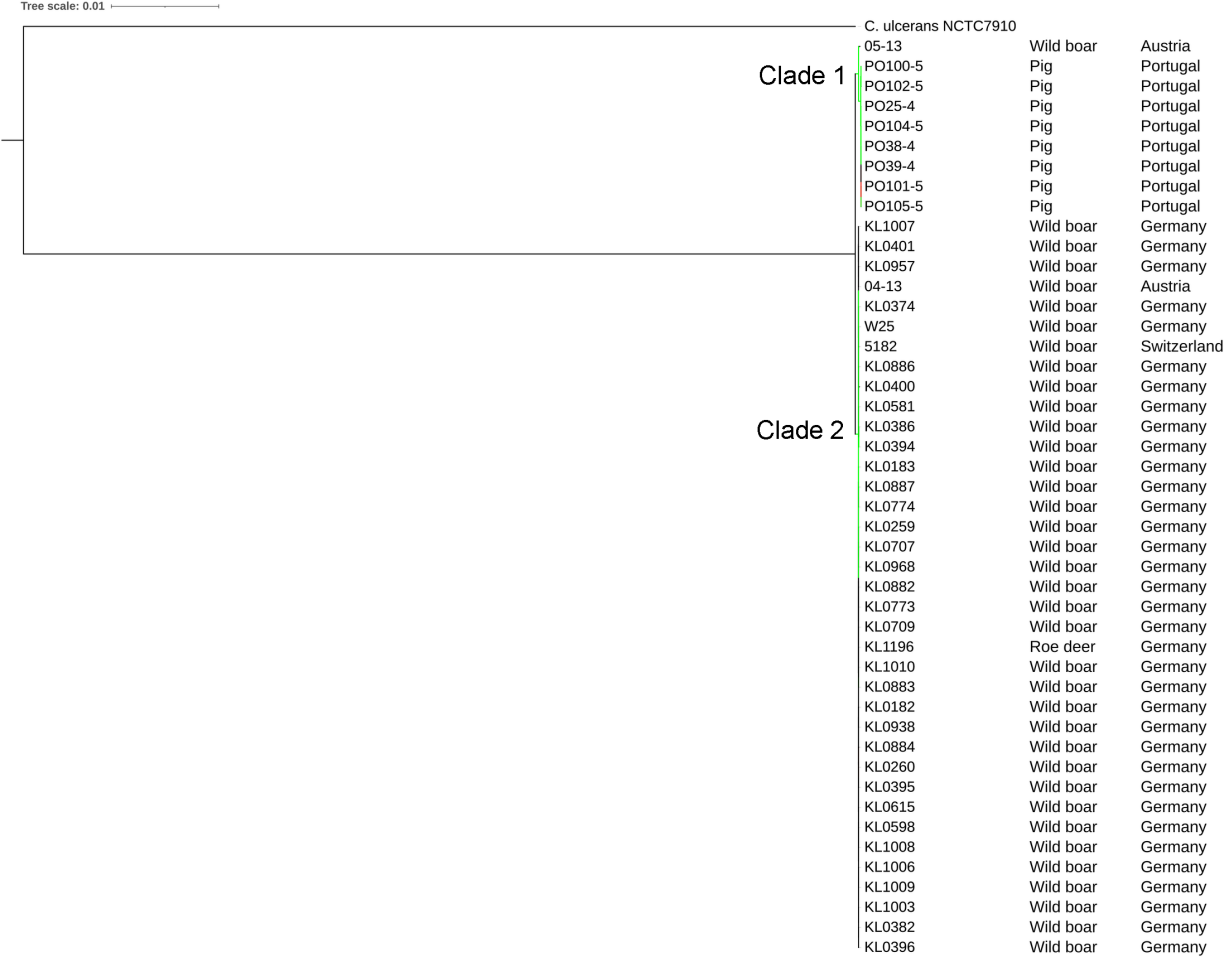
Phylogenomic tree of *Corynebacteriumsilvaticum* strains. The phylogeny was inferred using the Phylogenetic Tree service in PATRIC, which uses RAxML with 100 rounds of “rapid bootstrapping”, and the codon sequences from 1,000 single-copy shared genes. Bootstrap values are represented by a color scale from red (70%) to green (100%). *Corynebacterium ulcerans* NCTC7910 was used as an outgroup.

The strains were represented by 10 clusters, with strains from Germany in seven of them, being the most diverse. All strains from Portugal were in an exclusive cluster. Within strains from Austria, 05-13 had its own cluster, while 04-13 is part of a bigger cluster that includes strains from Germany and the single one from Switzerland (Fig. 3). The phylogenetic tree shows two clades. Clade 1 is monophyletic and has the strains from Portugal and strain 05-13 from Austria. Clade 2 has the remaining strains from Austria, Germany, and Switzerland (Fig. 4). The ANI values ranged from 99.7539 to 100% for all strains, and 99.9632 to 100% within the same clade. The difference between clades ranged from 0.2461 to 0.1729% (Data S7).

**Figure 5:**
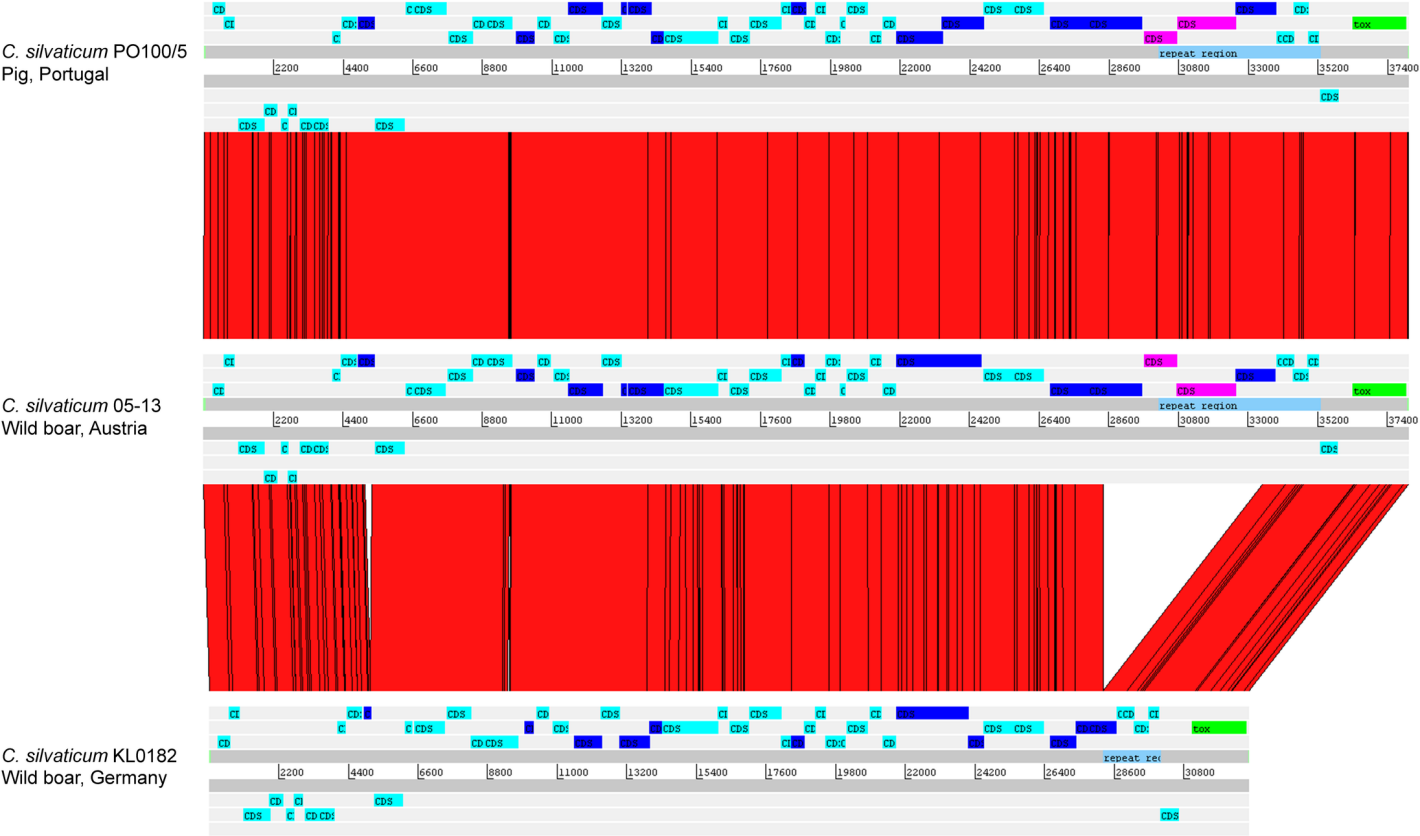
Alignment of *tox*^+^ prophages from *Corynebacterium silvaticum* strains. Coding sequences in dark blue are fragmented or fused in at least one genome. Coding sequences in pink are not present in strain KL0182^T^ prophage.

**Figure 6:**
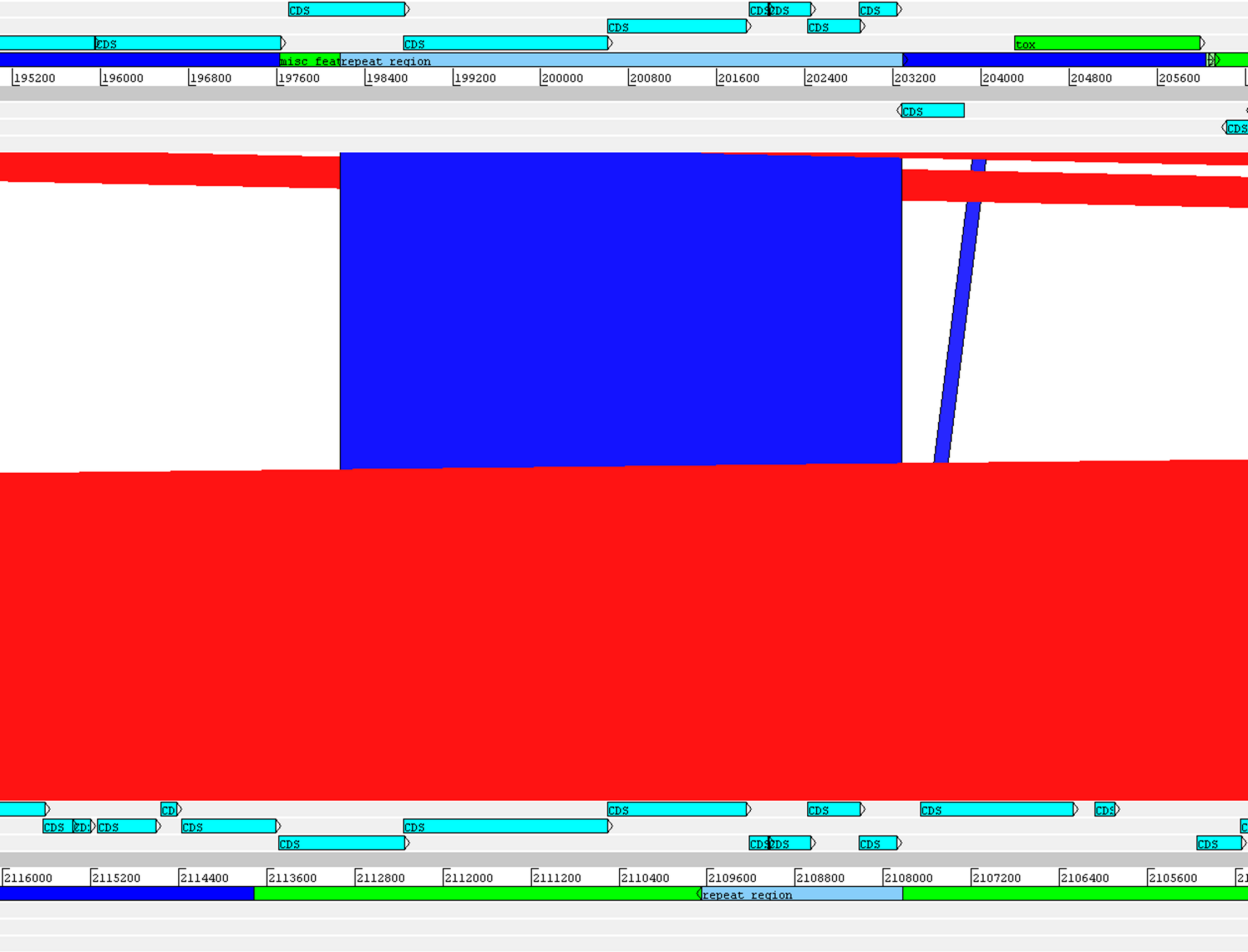
Alignment of part of the *tox*^+^ prophage from *Corynebacterium silvaticum* PO100/5 (top) and KL0182^T^ (bottom). The absent sequence in strain KL0182^T^ *tox*^+^ prophage is part of a repeat region and is in another part of the genome , inside genomic island GI 18 (bottom, green). Dark blue highlight in chromosome –prophage, green highlight in chromosome –genomic island.

The alignment of the *tox*^+^ prophages of PO100/5, 05-13 (Clade 1) and KL0182^T^ (Clade 2) shows nearly identical sequences for PO100/5 and 05-13, with a size of ∼38 Kb containing an additional 5.8 Kb sequence upstream the *tox* gene in comparison to KL0182^T^ (Wild boar, Germany, ∼32.8 Kb) (Fig. 5). The additional 5.8 Kb is a repeat and is also present in KL0182^T^ but in GI 18 rather than in *tox*^+^ prophage (Fig. 6). Mapping the sequencing reads of KL0182^T^ to its assembled genome and to PO100/5 genome confirmed that part of the prophage sequence is in another region of the genome (Data S14). Besides the insertion, coding sequences can be fragmented or fused when the *tox*^+^ prophage from PO100/5 and KL0182^T^ are compared (Fig. 5).

Across the 46 genomes, the pangenome, core genome, shared genome and singletons were 2,961, 2,227 (75.21%), 623 (21.04%) and 111 (3.75%) orthogroups, respectively (Data S15). The value of *α* was 0.968 (Data S16). The subsets of exclusively shared orthogroups were: 19 in *C. silvaticum* strains from Portugal, 25 in *C. silvaticum* strains from Portugal and strain 05-13 from Austria, and 36 from the remaining strains (Data S17 and S18). Of the 28 *Corynebacterium* virulence/niche factors described in the literature and identified in strains from Portugal (Table 2, S11 File), 19 are part of the core genome and eight are part of the shared genome (Data S15).

## Discussion

### Strains from Portugal and production of diphtheria toxin

All the Portuguese strains are monophyletic (Fig. 4), from the same cluster (Fig. 3), and nearly identical (Data S7), suggesting that they were derived from a single clone. The fact that the Portuguese strains have a most recent common ancestor with Austrian strain 05-13 suggests that the initial infection originated in Austria.

Eight virulence and niche factors identified in strains from Portugal are in the accessory genome (Data S14). *spaDEF* and *srtC* are part of a pilus cluster and are fragmented in different genomes. The absence of the *tox* gene in some strains is certainly an assembly artifact, since the strains lacking it were shown to have it (Dangel et al., 2019). The absence of sialidase (*nanH*) and serine protease (cpfrc_00397) in some strains could also be a sequence or assembly artifact, since they were detected in 45 out of 46 strains. The venom serine protease (*vsp1*) was found in all strains from Portugal and KL1008 from Germany, but the advantages for host colonization in comparison to other strains that lack the protein must be elucidated. Among the others, the niche factors Trypsin-like serine protease (Uniprot A0A5C5F2T7), *cwlH* (A0A5C5F4U0) and *rfpI* (A0A5F0A739) are part of the core genome (Data S15) and shared by all of the strains examined. These three proteins have been shown to be the most abundant extracellular proteins produced *in vitro* by *C. silvaticum* W25, representing 88.1, 2.2 and 1.3%, respectively (Möller et al., 2020b).

**Table 2:** Presence of niche and virulence factors of *Corynebacterium* in *C. silvaticum* strains from Portugal.

**Type**	**Gene**	**Product**	**Presence**	**Reference locus tag**	**Reference species**	**Reference**
Niche	–	*C. diphtheriae* DIP0733 homolog	Yes	CULC22_00609	Cul	Tauch & Burkovski (2015)
Niche	–	Secreted subtilisin-like serine protease	Yes	cpfrc_00397	Cp	Trost et al. (2010)
Niche	–	Secreted subtilisin-like serine protease	Yes, except in PO25/4	cpfrc_01634	Cp	Trost et al. (2010)
Niche	–	Secreted trypsin-like serine protease	No	cpfrc_00562	Cp	Trost et al. (2010)
Niche	–	Secreted SGNH-hydrolase	Yes	cpfrc_00536	Cp	Trost et al. (2010)
Niche	–	Trypsin-like serine protease	Yes	FIT55_05760, A0A5C5F2T7	Cs	Möller et al. (2020b)
Niche	*accD3*	Acyl-CoA carboxylase b-subunit involved in mycolic acid synthesis	Yes	cpfrc_01953	Cp	Trost et al. (2010)
Niche	*asa*	Ceramidase	No	jk1103	Cj	Tauch et al. (2005), Tauch & Burkovski (2015)
Niche	*che*	Cholesterol esterase	No	jk2054	Cj	Tauch et al. (2005), Tauch & Burkovski (2015)
Niche	*choE*	Cholesterol oxidase	No	jk0629	Cj	Tauch et al. (2005), Tauch & Burkovski (2015)
Niche	*cwlH*	Cell wall-associated hydrolase	Yes	CULC809_01521	Cul	Trost et al. (2011); Tauch & Burkovski (2015)
Niche	*dtsR1*	Acetyl-CoA carboxylase b-subunit involved in fatty acid synthesis	Yes	cpfrc_00492	Cp	Trost et al. (2010)
Niche	*dtsR2*	Acyl-CoA carboxylase b-subunit involved in mycolic acid synthesis	Yes	cpfrc_00491	Cp	Trost et al. (2010)
Niche	*endoE*	Endoglycosidase E (former corynebacterial protease CP40)	Yes	CULC809_01974	Cul	Trost et al. (2011)
Niche	*mdbA*	Thiol-disulfide oxidoreductase	Yes	DIP1880	Cd	Reardon-Robinson et al. (2015), Tauch & Burkovski (2015)
Niche	*nanH*	Sialidase (neuraminidase H)	Yes	CULC809_00434	Cul	Trost et al. (2011)
Niche	*nor*	Nitric oxide reductase	No	cpfrc_00128	Cp	Trost et al. (2010)
Niche	*nrpS1*	Nonribosomal peptide synthetase 1	Yes	cpfrc_00565	Cp	Trost et al. (2010)
Niche	*nrpS2*	Nonribosomal peptide synthetase 2	No	cpfrc_00180	Cp	Trost et al. (2010)
Niche	*rhuM*	RhuM-like protein	No	CulFRC58_0285	Cul	Trost et al. (2011); Weerasekera et al. (2019)
Niche	*rpfA*	Resuscitation-promoting factor A (muralytic enzyme)	Yes	cpfrc_00594	Cp	Trost et al. (2010)
Niche	*rpfB*	Resuscitation-promoting factor B (muralytic enzyme)	Yes	cpfrc_00679	Cp	Trost et al. (2010)
Niche	*rpfI*	Resuscitation-promoting factor-interacting protein	Yes	CULC809_01133	Cul	Trost et al. (2011); Tauch & Burkovski (2015)
Niche	*spaB*	Surface-anchored protein (minor pilus subunit)	Yes	CULC809_01980	Cul	Trost et al. (2011); Tauch & Burkovski (2015)
Niche	*spaC*	Surface-anchored protein (tip pilus protein)	Pseudogene, no CWSS	CULC809_01979	Cul	Trost et al. (2011); Tauch & Burkovski (2015)
Niche	*spaD*	Surface-anchored protein (major pilus subunit)	Pseudogene, no CWSS	CULC809_01952	Cul	Trost et al. (2011); Tauch & Burkovski (2015)
Niche	*spaE*	Surface-anchored protein (minor pilus subunit)	Pseudogene, no SP	CULC809_01950	Cul	Trost et al. (2011); Tauch & Burkovski (2015)
Niche	*spaF*	Surface-anchored protein (tip pilus protein)	Pseudogene, no SP	CULC809_01949	Cul	Trost et al. (2011); Tauch & Burkovski (2015)
Niche	*srtA*	Sortase A	Yes	CULC809_01981	Cul	Trost et al. (2011); Tauch & Burkovski (2015)
Niche	*srtB*	Sortase B	Yes	CULC809_01953	Cul	Trost et al. (2011); Tauch & Burkovski (2015)
Niche	*srtC*	Sortase C	Yes	CULC809_01951	Cul	Trost et al. (2011); Tauch & Burkovski (2015)
Niche	*tspA*	Trypsin-like serine protease	No	CULC809_01848	Cul	Trost et al. (2011)
Niche	*vsp1*	Venom serine protease	Yes	CULC809_00509	Cul	Trost et al. (2011)
Niche	*vsp2*	Venom serine protease	Yes	CULC809_01964	Cul	Trost et al. (2011)
Virulence	*pld*	Phospholipase D	Yes	ET810_03855	Cs	Trost et al. (2011); Tauch & Burkovski (2015)
Virulence	*rbp*	Shiga-like ribosome-binding protein	No	CULC809_00177	Cul	Trost et al. (2011); Tauch & Burkovski (2015)
Virulence	*tox*	Diphtheria toxin	Yes	DIP0222	Cd	Tauch & Burkovski (2015)
Virulence	*relA*	Guanosine-3′, 5′-bis(diphosphate) 3′-pyrophosphohydrolase / GTP pyrophosphokinase, (p)ppGpp synthetase II	Yes	–	–	Abricate
Virulence	*ideR*	Iron-dependent repressor IdeR/DtxR	Yes	–	–	Abricate
Virulence	*ureB*	Urease alpha subunit	Yes	–	–	Abricate
Resistance	*rpoB2*	Rifampin-resistant beta-subunit of RNA polymerase	Yes	–	–	Abricate
Resistance	*mtrA*	Two component system response regulator MtrA	Yes	–	–	Abricate
Resistance	*rbpA*	RNA-polymerase binding protein which confers resistance to rifampin	Yes	–	–	Abricate

**Notes.**

CWSS cell wall sorting signal SP signal peptide

*C. silvaticum* was described as non-toxigenic *tox* gene-bearing (Dangel et al., 2020). An insertion of two guanines in *tox* caused a frameshift and the pseudogenization of this gene (Möller et al., 2020a). However, the strains PO100/5 from Portugal and 05-13 lack the insertion and therefore could produce the toxin (Viana et al., 2020). We confirmed that all eight strains from Portugal lack that insertion that knocks out this gene (Fig. 2, Data S13) and are probable DT producers. The production of DT in strains 4-13 and 05-13 from Austria was inferred by RT-qPCR (Schaeffer et al., 2020), targeting subunits A and B of the DT (Mothershed et al., 2002). However, strain 04-13 has the typical frameshift in *tox*. The primers binding sites start from position 312, while the frameshift occurs in position 44 (Fig. 2, Data S13), so the frameshift cannot not be detected. It was suggested that DT production must be confirmed on *tox*-positive isolates by an Elek test, due to the description of *tox*-positive and Elek-negative strains (Schuhegger et al., 2008; Berger et al., 2014). This shows a limitation of the current RT-qPCR for detection of DT, and that 04-13 probably does not produce the toxin.

DT is a virulence factor because it only works inside of host cells and damages them (Tauch & Burkovski, 2015), but that may not impact *C. silvaticum*’s host range in the wild. The species causes CLA in its known hosts (Dangel et al., 2020), a disease manifestation like the non-DT-producing *C. pseudotuberculosis* biovar ovis causes in goats and sheep (Dorella et al., 2006). CLA is related to the toxin Phospholipase D (*pld*) (Dorella et al., 2006) that is also produced by *C. silvaticum* (Dangel et al., 2020). Another possibility is that the DT is required only for the infection of as yet unidentified hosts. It has been hypothesized that the production of DT is required for *C. pseudotuberculosis* biovar equi to infect buffalo (Viana et al., 2017). If DT production is not required, this could relax the selective pressure to keep a functional *tox* gene and could lead to accumulation mutations that would result in the loss of function (Figs. 2 and 5) seen in some populations of Germany, Austria and Switzerland.

### Genome diversity of *C. silvaticum*

The phylogenetic tree showed that strains from Portugal and 05-13 from Austria formed Clade 1 and the remaining strains formed Clade 2 (Fig. 4). The two strains from Austria (04-13 and 05-13) are in different clades, suggesting different geographical origins. The strains from Portugal are in the same clade and nearly identical to 05-13, which suggests an Austrian origin.

Although the genomes had high ANI values, of at least 99.7% (Data S7), we could identify 10 clusters (Figs. 3 and 4). Strains from Germany had higher diversity, with strains in seven clusters, probably due to the higher number of samples and isolation from wild animals. All strains from Portugal had an exclusive cluster. As they were isolated from domestic pigs in two farms (Oliveira et al., 2014) this represents the spread of only one clone. More strains must be analyzed to assess the diversity of the species.

Across the 46 genomes, we identified a pangenome of 2,961 orthogroups, core genome of 2,227, 623 shared orthogroups, 111 singletons and an *α* of 0.968 (Data S5 and Data S15). The pangenome is nearly closed, which agrees with the high ANI values between the genomes. We identified exclusively shared orthogroups in strains from Portugal (19 orthogroups), Clade 1 (27 orthogroups), and Clade 2 (36 orthogroups) (Data S17 and S18). Those genes are probably not involved in the manifestation of the disease as it is the same manifestation (CL) in the known hosts but could be required for survival outside of the host.

With the presence of *C. silvaticum* in wild and domestic animals, cytotoxicity to human epithelial cells (Möller et al., 2021) and the probable production of DT, this species has the potential to cause zoonosis and diphtheria. Human transmission could occur *via* occupational exposure, as seen in *C. pseudotuberculosis* (Dorella et al., 2006). In this context, we identified 10 clusters (Fig. 3), exclusively shared genes of clades and Portuguese strains (Data S17 and S18), and the exclusive STs from Portugal (Data S9). This information can be applied to the identification and epidemiology of *C. silvaticum*.

## Conclusions

In *C. silvaticum*, Clade 1 includes strains that have the potential to produce DT, which is missing in Clade 2 (non-DT-producing). Both clades can be identified by genes that, while probably not important for their interactions within the host environment, could play a role in survival in the environment. Portuguese strains are monophyletic, nearly identical, form a unique cluster and probably produce DT. We showed that the species has the potential to cause zoonosis and diphtheria, and genome clusters, STs and exclusive genes that can be applied to its epidemiology.

##  Supplemental Information

10.7717/peerj.14895/supp-1 Supplemental Information 1 Genome samples of* Corynebacteriumsilvaticum*, *C. ulcerans* and *C. pseudotuberculosis.*

10.7717/peerj.14895/supp-2 Supplemental Information 2 In-house scripts for orthologous gene groups analysis

10.7717/peerj.14895/supp-3 Supplemental Information 3 Taxonomic classification of genomes using the Type Strain Genome Server

10.7717/peerj.14895/supp-4 Supplemental Information 4 Insertion sequences of *Corynebacterium silvaticum* strains from Portugal

10.7717/peerj.14895/supp-5 Supplemental Information 5 Prophages of *Corynebacterium silvaticum* strains from Portugal

10.7717/peerj.14895/supp-6 Supplemental Information 6 Crispr-Cas systems of *Corynebacterium silvaticum* strains from Portugal

10.7717/peerj.14895/supp-7 Supplemental Information 7 Average Nucleotide values of *Corynebacterium silvaticum* strains

10.7717/peerj.14895/supp-8 Supplemental Information 8 Genomic islands of *Corynebacterium silvaticum* strains from Portugal

10.7717/peerj.14895/supp-9 Supplemental Information 9 Multilocus Sequence Types of 146 *Corynebacterium silvaticum* strains

10.7717/peerj.14895/supp-10 Supplemental Information 10 Presence of virulence factors in *Corynebacterium silvaticum* strains from Portugal, predicted using Abricate

10.7717/peerj.14895/supp-11 Supplemental Information 11 Presence of known *Corynebacterium* virulence factors from literature in *C. silvaticum* strains from Portugal, predicted using PATRIC’s Proteome Comparison Tool

10.7717/peerj.14895/supp-12 Supplemental Information 12 Alignment of the DT sequences from *Corynebacterium silvaticum* strains, *C. ulcerans* 0102, *C. pseudotuberculosis* 31 and *C. diphtheriae* NCTC 13129

10.7717/peerj.14895/supp-13 Supplemental Information 13 Alignment of the *tox* gene sequences from *Corynebacterium silvaticum* strains, *C. ulcerans* 0102, *C. pseudotuberculosis* 31 and *C. diphtheriae* NCTC 13129, showing the flanking region of the primers used for RT-qPCR

10.7717/peerj.14895/supp-14 Supplemental Information 14 Mapping of strain KL0182^T^ sequencing reads to the *tox^+^* prophage in its own genome (A) and PO100/5 genome (B). The region in PO100/5 with no read mapping is missing in KL0182^T^*tox^+^* prophage

10.7717/peerj.14895/supp-15 Supplemental Information 15 Orthologs in the pangenome, core genome, shared genome and singletons of 46 *Corynebacterium silvaticum* genomes identified using OrthoFinder

10.7717/peerj.14895/supp-16 Supplemental Information 16 Pangenome statistics of *Corynebacterium silvaticum*

10.7717/peerj.14895/supp-17 Supplemental Information 17 Exclusively shared orthologous groups across groups of strains

10.7717/peerj.14895/supp-18 Supplemental Information 18 Annotation of exclusively shared orthologous groups across groups of strains

## Additional Information and Declarations

### Data Availability

The following information was supplied regarding data availability: The scripts used for analysis of OrthoFinder output are available in the Supplementary File.
