# Supplementary material for: Analysis of Corynebacterium silvaticum genomes from Portugal reveals a single cluster and a clade suggested to produce diphtheria toxin
Source: PeerJ. 2023 Mar 9;11:e14895. doi: 10.7717/peerj.14895 (PMC10008321; doi:10.7717/peerj.14895)
Supplement: Supplemental Information 3 [file peerj-11-14895-s003.pdf]

TYPE (STRAIN) GENOME SERVER

JOB SUMMARY

PRINT DATE:	2021-08-11 20:36:13 +0200
JOB ID:	cfd10836-3505-4425-b6fd-ef9f3e3075fe
RESULT PAGE:	https://tygs.dsmz.de/user_results/show?guid=cfd10836-3505-4425-b6fd-ef9f3e3075fe

Table 1: Phylogenies

Publication-ready versions of both the genome-scale GBDP tree and the 16S rRNA gene sequence tree can be customized and exported either in SVG (vector graphic) or PNG format from within the phylogeny viewers in your TYGS result page. For publications the **SVG format is recommended** because it is lossless, always keeps its high resolution and can also be easily converted to other popular formats such as PDF or EPS. Please follow the link provided above!

Table 2: Identification

The below list contains the result of the TYGS species identification routine.

Explanation of remarks that might occur in the below table:

remark [R1]: The TYGS type strain database is automatically updated on an almost daily basis. However, if a particular type strain genome is not available in the TYGS database, this can have several reasons which are detailed in the FAQ. You can request an extended 16S rRNA gene analysis via the 16S tree viewer found in your result page to detect **not yet genome-sequenced** type strains relevant for your study.

remark [R2]: > 70% dDDH value (formula d_4) and (almost) minimal dDDH values for gene-content formulae d_0 and d_6 indicate a potentially unreliable identification result and should thus be checked via the 16S rRNA gene sequence similarity. Such strong deviations can, in principle, be caused by sequence contamination.

remark [R3]: G+C content difference of > 1 % indicates a potentially unreliable identification result because within species G+C content varies no more than 1 %, if computed from genome sequences (PMID: 24505073).

Strain	Conclusion	Identification result	Remark
**'Cs5182'**	belongs to known species	*Corynebacterium silvaticum*	
**'CsPO25-4_unicycler'**	belongs to known species	*Corynebacterium silvaticum*	
**'CsPO38-4_unicycler'**	belongs to known species	*Corynebacterium silvaticum*	
**'CsPO39-4_unicycler'**	belongs to known species	*Corynebacterium silvaticum*	
**'CsPO100-5_unicycler'**	belongs to known species	*Corynebacterium silvaticum*	
**'CsPO101-5_unicycler'**	belongs to known species	*Corynebacterium silvaticum*	
**'CsPO102-5_unicycler'**	belongs to known species	*Corynebacterium silvaticum*	
**'CsPO104-5_unicycler'**	belongs to known species	*Corynebacterium silvaticum*	
**'CsPO105-5_unicycler'**	belongs to known species	*Corynebacterium silvaticum*	

Table 3: Pairwise comparisons of user genomes vs. type-strain genomes

The following table contains the pairwise dDDH values between your user genomes and the selected type-strain genomes. The dDDH values are provided along with their confidence intervals (C.I.) for the three different GBDP formulas:

- formula d_0 (a.k.a. GGDC formula 1): length of all HSPs divided by total genome length
- formula d_4 (a.k.a. GGDC formula 2): sum of all identities found in HSPs divided by overall HSP length
- formula d_6 (a.k.a. GGDC formula 3): sum of all identities found in HSPs divided by total genome length

Note: Formula d_4 is independent of genome length and is thus robust against the use of incomplete draft genomes. For other reasons for preferring formula d_4, see the FAQ.

Query	Subject	d_0	C.I. d_0	d_4	C.I. d_4	d_6	C.I. d_6	Diff. G+C Percent
**'CsPO38-4_unicycler.fasta'**	**'CsPO102-5_unicycler.fasta'**	100.0	[100.0 - 100.0]	100.0	[99.9 - 100.0]	100.0	[100.0 - 100.0]	0.0
**'Cs5182.fasta'**	*Corynebacterium silvaticum* KL0182	100.0	[100.0 - 100.0]	100.0	[100.0 - 100.0]	100.0	[100.0 - 100.0]	0.01
**'CsPO25-4_unicycler.fasta'**	**'CsPO104-5_unicycler.fasta'**	100.0	[100.0 - 100.0]	100.0	[100.0 - 100.0]	100.0	[100.0 - 100.0]	0.0
**'CsPO38-4_unicycler.fasta'**	**'CsPO100-5_unicycler.fasta'**	100.0	[100.0 - 100.0]	100.0	[99.9 - 100.0]	100.0	[100.0 - 100.0]	0.0
**'CsPO39-4_unicycler.fasta'**	**'CsPO104-5_unicycler.fasta'**	100.0	[100.0 - 100.0]	100.0	[100.0 - 100.0]	100.0	[100.0 - 100.0]	0.0
**'CsPO38-4_unicycler.fasta'**	**'CsPO39-4_unicycler.fasta'**	100.0	[100.0 - 100.0]	100.0	[100.0 - 100.0]	100.0	[100.0 - 100.0]	0.0
**'CsPO101-5_unicycler.fasta'**	**'CsPO102-5_unicycler.fasta'**	100.0	[100.0 - 100.0]	100.0	[99.9 - 100.0]	100.0	[100.0 - 100.0]	0.0
**'CsPO100-5_unicycler.fasta'**	**'CsPO105-5_unicycler.fasta'**	100.0	[100.0 - 100.0]	100.0	[99.9 - 100.0]	100.0	[100.0 - 100.0]	0.0
**'CsPO25-4_unicycler.fasta'**	**'CsPO39-4_unicycler.fasta'**	100.0	[100.0 - 100.0]	100.0	[100.0 - 100.0]	100.0	[100.0 - 100.0]	0.0
**'CsPO39-4_unicycler.fasta'**	**'CsPO100-5_unicycler.fasta'**	100.0	[100.0 - 100.0]	100.0	[99.9 - 100.0]	100.0	[100.0 - 100.0]	0.0
**'CsPO25-4_unicycler.fasta'**	**'CsPO105-5_unicycler.fasta'**	100.0	[100.0 - 100.0]	100.0	[100.0 - 100.0]	100.0	[100.0 - 100.0]	0.0
**'CsPO25-4_unicycler.fasta'**	**'CsPO102-5_unicycler.fasta'**	100.0	[100.0 - 100.0]	100.0	[99.9 - 100.0]	100.0	[100.0 - 100.0]	0.0
**'CsPO102-5_unicycler.fasta'**	**'CsPO104-5_unicycler.fasta'**	100.0	[100.0 - 100.0]	100.0	[99.9 - 100.0]	100.0	[100.0 - 100.0]	0.0
**'CsPO38-4_unicycler.fasta'**	**'CsPO104-5_unicycler.fasta'**	100.0	[100.0 - 100.0]	100.0	[100.0 - 100.0]	100.0	[100.0 - 100.0]	0.0
**'CsPO100-5_unicycler.fasta'**	**'CsPO102-5_unicycler.fasta'**	100.0	[100.0 - 100.0]	100.0	[100.0 - 100.0]	100.0	[100.0 - 100.0]	0.0
**'CsPO38-4_unicycler.fasta'**	**'CsPO101-5_unicycler.fasta'**	100.0	[100.0 - 100.0]	100.0	[100.0 - 100.0]	100.0	[100.0 - 100.0]	0.0
**'CsPO39-4_unicycler.fasta'**	**'CsPO105-5_unicycler.fasta'**	100.0	[100.0 - 100.0]	100.0	[100.0 - 100.0]	100.0	[100.0 - 100.0]	0.0
**'CsPO102-5_unicycler.fasta'**	**'CsPO105-5_unicycler.fasta'**	100.0	[100.0 - 100.0]	100.0	[99.9 - 100.0]	100.0	[100.0 - 100.0]	0.0
**'CsPO39-4_unicycler.fasta'**	**'CsPO102-5_unicycler.fasta'**	100.0	[100.0 - 100.0]	100.0	[99.9 - 100.0]	100.0	[100.0 - 100.0]	0.0
**'CsPO25-4_unicycler.fasta'**	**'CsPO100-5_unicycler.fasta'**	100.0	[100.0 - 100.0]	100.0	[99.9 - 100.0]	100.0	[100.0 - 100.0]	0.0
**'CsPO100-5_unicycler.fasta'**	**'CsPO104-5_unicycler.fasta'**	100.0	[100.0 - 100.0]	100.0	[99.9 - 100.0]	100.0	[100.0 - 100.0]	0.0
**'CsPO104-5_unicycler.fasta'**	**'CsPO105-5_unicycler.fasta'**	100.0	[100.0 - 100.0]	100.0	[100.0 - 100.0]	100.0	[100.0 - 100.0]	0.0

Query	Subject	d_0	C.I. d_0	d_4	C.I. d_4	d_6	C.I. d_6	Diff. G+C Percent
**'CsPO100-5_unicycler.fasta'**	**'CsPO101-5_unicycler.fasta'**	100.0	[100.0 - 100.0]	100.0	[99.9 - 100.0]	100.0	[100.0 - 100.0]	0.0
**'CsPO25-4_unicycler.fasta'**	**'CsPO101-5_unicycler.fasta'**	100.0	[100.0 - 100.0]	100.0	[100.0 - 100.0]	100.0	[100.0 - 100.0]	0.0
**'CsPO101-5_unicycler.fasta'**	**'CsPO105-5_unicycler.fasta'**	100.0	[100.0 - 100.0]	100.0	[100.0 - 100.0]	100.0	[100.0 - 100.0]	0.0
**'CsPO25-4_unicycler.fasta'**	**'CsPO38-4_unicycler.fasta'**	100.0	[100.0 - 100.0]	100.0	[100.0 - 100.0]	100.0	[100.0 - 100.0]	0.0
**'CsPO101-5_unicycler.fasta'**	**'CsPO104-5_unicycler.fasta'**	100.0	[100.0 - 100.0]	100.0	[100.0 - 100.0]	100.0	[100.0 - 100.0]	0.0
**'CsPO38-4_unicycler.fasta'**	**'CsPO105-5_unicycler.fasta'**	100.0	[100.0 - 100.0]	100.0	[100.0 - 100.0]	100.0	[100.0 - 100.0]	0.0
**'CsPO39-4_unicycler.fasta'**	**'CsPO101-5_unicycler.fasta'**	100.0	[100.0 - 100.0]	100.0	[100.0 - 100.0]	100.0	[100.0 - 100.0]	0.0
**'Cs5182.fasta'**	**'CsPO102-5_unicycler.fasta'**	99.7	[99.4 - 99.9]	98.6	[97.9 - 99.1]	99.8	[99.7 - 99.9]	0.02
**'Cs5182.fasta'**	**'CsPO39-4_unicycler.fasta'**	99.7	[99.4 - 99.9]	98.6	[97.9 - 99.1]	99.8	[99.7 - 99.9]	0.02
**'CsPO39-4_unicycler.fasta'**	*Corynebacterium silvaticum* KL0182	99.7	[99.4 - 99.9]	98.6	[97.9 - 99.0]	99.8	[99.7 - 99.9]	0.03
**'Cs5182.fasta'**	**'CsPO25-4_unicycler.fasta'**	99.7	[99.4 - 99.9]	98.6	[97.9 - 99.1]	99.8	[99.7 - 99.9]	0.02
**'CsPO38-4_unicycler.fasta'**	*Corynebacterium silvaticum* KL0182	99.7	[99.4 - 99.9]	98.6	[97.9 - 99.0]	99.8	[99.7 - 99.9]	0.03
**'CsPO25-4_unicycler.fasta'**	*Corynebacterium silvaticum* KL0182	99.7	[99.4 - 99.9]	98.6	[97.9 - 99.0]	99.8	[99.7 - 99.9]	0.03
**'CsPO104-5_unicycler.fasta'**	*Corynebacterium silvaticum* KL0182	99.7	[99.4 - 99.9]	98.6	[97.9 - 99.0]	99.8	[99.7 - 99.9]	0.03
**'Cs5182.fasta'**	**'CsPO38-4_unicycler.fasta'**	99.7	[99.4 - 99.9]	98.6	[97.9 - 99.1]	99.8	[99.7 - 99.9]	0.02
**'CsPO105-5_unicycler.fasta'**	*Corynebacterium silvaticum* KL0182	99.7	[99.4 - 99.9]	98.6	[97.9 - 99.0]	99.8	[99.7 - 99.9]	0.03
**'Cs5182.fasta'**	**'CsPO105-5_unicycler.fasta'**	99.7	[99.4 - 99.9]	98.6	[97.9 - 99.1]	99.8	[99.7 - 99.9]	0.02
**'Cs5182.fasta'**	**'CsPO100-5_unicycler.fasta'**	99.7	[99.4 - 99.9]	98.6	[97.9 - 99.1]	99.8	[99.7 - 99.9]	0.02
**'CsPO102-5_unicycler.fasta'**	*Corynebacterium silvaticum* KL0182	99.7	[99.4 - 99.9]	98.6	[97.9 - 99.0]	99.8	[99.7 - 99.9]	0.03
**'Cs5182.fasta'**	**'CsPO104-5_unicycler.fasta'**	99.7	[99.4 - 99.9]	98.6	[97.9 - 99.1]	99.8	[99.7 - 99.9]	0.02
**'CsPO100-5_unicycler.fasta'**	*Corynebacterium silvaticum* KL0182	99.7	[99.4 - 99.9]	98.6	[97.9 - 99.0]	99.8	[99.7 - 99.9]	0.03
**'CsPO101-5_unicycler.fasta'**	*Corynebacterium silvaticum* KL0182	99.7	[99.4 - 99.9]	98.6	[97.9 - 99.0]	99.8	[99.7 - 99.9]	0.03
**'Cs5182.fasta'**	**'CsPO101-5_unicycler.fasta'**	99.7	[99.4 - 99.9]	98.6	[97.9 - 99.1]	99.8	[99.7 - 99.9]	0.02
**'CsPO25-4_unicycler.fasta'**	*Corynebacterium ulcerans* NCTC 7910	91.8	[88.8 - 94.0]	41.0	[38.5 - 43.5]	81.6	[78.2 - 84.5]	1.11
**'CsPO38-4_unicycler.fasta'**	*Corynebacterium ulcerans* NCTC 7910	91.8	[88.8 - 94.0]	41.0	[38.5 - 43.5]	81.6	[78.2 - 84.5]	1.11
**'CsPO39-4_unicycler.fasta'**	*Corynebacterium ulcerans* NCTC 7910	91.8	[88.8 - 94.0]	41.0	[38.5 - 43.5]	81.6	[78.2 - 84.5]	1.11
**'CsPO101-5_unicycler.fasta'**	*Corynebacterium ulcerans* NCTC 7910	91.8	[88.8 - 94.0]	41.0	[38.5 - 43.5]	81.6	[78.2 - 84.5]	1.11

Query	Subject	d_0	C.I. d_0	d_4	C.I. d_4	d_6	C.I. d_6	Diff. G+C Percent
**'CsPO104-5_unicycler.fasta'**	*Corynebacterium ulcerans* NCTC 7910	91.8	[88.8 - 94.0]	41.0	[38.5 - 43.5]	81.6	[78.2 - 84.5]	1.11
**'Cs5182.fasta'**	*Corynebacterium ulcerans* NCTC 7910	91.8	[88.9 - 94.1]	40.9	[38.4 - 43.5]	81.6	[78.2 - 84.5]	1.12
**'CsPO100-5_unicycler.fasta'**	*Corynebacterium ulcerans* NCTC 7910	91.8	[88.8 - 94.0]	40.9	[38.5 - 43.5]	81.6	[78.2 - 84.5]	1.11
**'CsPO105-5_unicycler.fasta'**	*Corynebacterium ulcerans* NCTC 7910	91.8	[88.8 - 94.0]	40.9	[38.5 - 43.5]	81.6	[78.2 - 84.5]	1.11
**'CsPO102-5_unicycler.fasta'**	*Corynebacterium ulcerans* NCTC 7910	91.8	[88.8 - 94.0]	40.9	[38.5 - 43.5]	81.6	[78.2 - 84.5]	1.11
**'CsPO102-5_unicycler.fasta'**	*Corynebacterium pseudotuberculosis* ATCC 19410	83.1	[79.3 - 86.3]	28.5	[26.1 - 31.0]	66.0	[62.7 - 69.3]	2.24
**'CsPO105-5_unicycler.fasta'**	*Corynebacterium pseudotuberculosis* ATCC 19410	83.1	[79.3 - 86.3]	28.5	[26.1 - 31.0]	66.0	[62.7 - 69.3]	2.24
**'CsPO38-4_unicycler.fasta'**	*Corynebacterium pseudotuberculosis* ATCC 19410	83.1	[79.3 - 86.3]	28.5	[26.1 - 31.0]	66.0	[62.7 - 69.3]	2.24
**'CsPO25-4_unicycler.fasta'**	*Corynebacterium pseudotuberculosis* DSM 20689	83.2	[79.4 - 86.4]	28.5	[26.1 - 31.0]	66.1	[62.7 - 69.4]	2.23
**'CsPO100-5_unicycler.fasta'**	*Corynebacterium pseudotuberculosis* ATCC 19410	83.1	[79.3 - 86.3]	28.5	[26.1 - 31.0]	66.0	[62.7 - 69.3]	2.24
**'CsPO101-5_unicycler.fasta'**	*Corynebacterium pseudotuberculosis* DSM 20689	83.2	[79.4 - 86.4]	28.5	[26.1 - 31.0]	66.1	[62.7 - 69.4]	2.23
**'CsPO105-5_unicycler.fasta'**	*Corynebacterium pseudotuberculosis* DSM 20689	83.2	[79.4 - 86.4]	28.5	[26.1 - 31.0]	66.1	[62.7 - 69.4]	2.23
**'CsPO100-5_unicycler.fasta'**	*Corynebacterium pseudotuberculosis* DSM 20689	83.2	[79.4 - 86.5]	28.5	[26.1 - 31.0]	66.1	[62.7 - 69.4]	2.23
**'CsPO39-4_unicycler.fasta'**	*Corynebacterium pseudotuberculosis* ATCC 19410	83.1	[79.3 - 86.3]	28.5	[26.1 - 31.0]	66.0	[62.7 - 69.3]	2.24
**'CsPO101-5_unicycler.fasta'**	*Corynebacterium pseudotuberculosis* ATCC 19410	83.1	[79.3 - 86.3]	28.5	[26.1 - 31.0]	66.0	[62.7 - 69.3]	2.24
**'CsPO104-5_unicycler.fasta'**	*Corynebacterium pseudotuberculosis* ATCC 19410	83.1	[79.3 - 86.3]	28.5	[26.1 - 31.0]	66.0	[62.7 - 69.3]	2.24
**'CsPO102-5_unicycler.fasta'**	*Corynebacterium pseudotuberculosis* DSM 20689	83.2	[79.4 - 86.5]	28.5	[26.1 - 31.0]	66.1	[62.7 - 69.4]	2.23
**'CsPO25-4_unicycler.fasta'**	*Corynebacterium pseudotuberculosis* ATCC 19410	83.1	[79.3 - 86.3]	28.5	[26.1 - 31.0]	66.0	[62.7 - 69.3]	2.24
**'Cs5182.fasta'**	*Corynebacterium pseudotuberculosis* ATCC 19410	82.9	[79.1 - 86.2]	28.5	[26.2 - 31.0]	66.0	[62.6 - 69.2]	2.25
**'CsPO38-4_unicycler.fasta'**	*Corynebacterium pseudotuberculosis* DSM 20689	83.2	[79.4 - 86.4]	28.5	[26.1 - 31.0]	66.1	[62.7 - 69.4]	2.23
**'Cs5182.fasta'**	*Corynebacterium pseudotuberculosis* DSM 20689	83.0	[79.2 - 86.3]	28.5	[26.2 - 31.0]	66.0	[62.6 - 69.3]	2.25
**'CsPO39-4_unicycler.fasta'**	*Corynebacterium pseudotuberculosis* DSM 20689	83.2	[79.4 - 86.4]	28.5	[26.1 - 31.0]	66.1	[62.7 - 69.4]	2.23

Query	Subject	d_0	C.I. d_0	d_4	C.I. d_4	d_6	C.I. d_6	Diff. G+C Percent
**'CsPO104-5_unicycler.fasta'**	*Corynebacterium pseudotuberculosis* DSM 20689	83.2	[79.4 - 86.4]	28.5	[26.1 - 31.0]	66.1	[62.7 - 69.4]	2.23
**'CsPO38-4_unicycler.fasta'**	*Corynebacterium resistens* DSM 45100	13.1	[10.4 - 16.4]	26.4	[24.0 - 28.9]	13.5	[11.1 - 16.2]	2.67
**'CsPO100-5_unicycler.fasta'**	*Corynebacterium resistens* DSM 45100	13.1	[10.4 - 16.4]	26.4	[24.0 - 28.9]	13.5	[11.1 - 16.2]	2.67
**'CsPO104-5_unicycler.fasta'**	*Corynebacterium resistens* DSM 45100	13.1	[10.4 - 16.4]	26.4	[24.0 - 28.9]	13.5	[11.1 - 16.2]	2.67
**'CsPO102-5_unicycler.fasta'**	*Corynebacterium resistens* DSM 45100	13.1	[10.4 - 16.4]	26.4	[24.0 - 28.9]	13.5	[11.1 - 16.2]	2.67
**'CsPO101-5_unicycler.fasta'**	*Corynebacterium resistens* DSM 45100	13.1	[10.4 - 16.4]	26.4	[24.0 - 28.9]	13.5	[11.1 - 16.2]	2.67
**'CsPO105-5_unicycler.fasta'**	*Corynebacterium resistens* DSM 45100	13.1	[10.4 - 16.4]	26.4	[24.0 - 28.9]	13.5	[11.1 - 16.2]	2.67
**'CsPO25-4_unicycler.fasta'**	*Corynebacterium resistens* DSM 45100	13.1	[10.4 - 16.4]	26.4	[24.0 - 28.9]	13.5	[11.1 - 16.2]	2.67
**'CsPO39-4_unicycler.fasta'**	*Corynebacterium resistens* DSM 45100	13.1	[10.4 - 16.4]	26.4	[24.0 - 28.9]	13.5	[11.1 - 16.2]	2.67
**'Cs5182.fasta'**	*Corynebacterium resistens* DSM 45100	13.1	[10.4 - 16.4]	24.8	[22.5 - 27.3]	13.5	[11.1 - 16.3]	2.66
**'Cs5182.fasta'**	*Corynebacterium vitaeruminis* DSM 20294	13.4	[10.7 - 16.8]	24.4	[22.1 - 26.9]	13.8	[11.4 - 16.6]	11.09
**'Cs5182.fasta'**	*Corynebacterium kutscheri* DSM 20755	13.1	[10.4 - 16.4]	24.0	[21.7 - 26.4]	13.5	[11.1 - 16.2]	7.97
**'CsPO102-5_unicycler.fasta'**	*Corynebacterium kutscheri* DSM 20755	13.1	[10.4 - 16.4]	23.9	[21.6 - 26.4]	13.5	[11.1 - 16.2]	7.96
**'CsPO100-5_unicycler.fasta'**	*Corynebacterium kutscheri* DSM 20755	13.1	[10.4 - 16.4]	23.9	[21.6 - 26.4]	13.5	[11.1 - 16.2]	7.96
**'CsPO104-5_unicycler.fasta'**	*Corynebacterium kutscheri* DSM 20755	13.1	[10.4 - 16.4]	23.9	[21.6 - 26.4]	13.5	[11.1 - 16.2]	7.96
**'CsPO105-5_unicycler.fasta'**	*Corynebacterium kutscheri* DSM 20755	13.1	[10.4 - 16.4]	23.9	[21.6 - 26.4]	13.5	[11.1 - 16.2]	7.96
**'CsPO39-4_unicycler.fasta'**	*Corynebacterium kutscheri* DSM 20755	13.1	[10.4 - 16.4]	23.9	[21.6 - 26.4]	13.5	[11.1 - 16.2]	7.96
**'CsPO101-5_unicycler.fasta'**	*Corynebacterium kutscheri* DSM 20755	13.1	[10.4 - 16.4]	23.9	[21.6 - 26.4]	13.5	[11.1 - 16.2]	7.96
**'CsPO38-4_unicycler.fasta'**	*Corynebacterium kutscheri* DSM 20755	13.1	[10.4 - 16.4]	23.9	[21.6 - 26.4]	13.5	[11.1 - 16.2]	7.96
**'CsPO25-4_unicycler.fasta'**	*Corynebacterium kutscheri* DSM 20755	13.1	[10.4 - 16.4]	23.9	[21.6 - 26.4]	13.5	[11.1 - 16.2]	7.96
**'CsPO105-5_unicycler.fasta'**	*Corynebacterium vitaeruminis* DSM 20294	13.5	[10.8 - 16.8]	23.8	[21.5 - 26.3]	13.9	[11.5 - 16.7]	11.11
**'CsPO39-4_unicycler.fasta'**	*Corynebacterium vitaeruminis* DSM 20294	13.5	[10.8 - 16.8]	23.8	[21.5 - 26.3]	13.9	[11.5 - 16.7]	11.11
**'CsPO25-4_unicycler.fasta'**	*Corynebacterium vitaeruminis* DSM 20294	13.5	[10.8 - 16.8]	23.8	[21.5 - 26.3]	13.9	[11.5 - 16.7]	11.11
**'CsPO100-5_unicycler.fasta'**	*Corynebacterium diphtheriae* subsp. *lausannense* CHUV2995	14.3	[11.5 - 17.7]	23.8	[21.5 - 26.2]	14.6	[12.2 - 17.5]	0.48
**'CsPO102-5_unicycler.fasta'**	*Corynebacterium diphtheriae* subsp. *lausannense* CHUV2995	14.3	[11.5 - 17.7]	23.8	[21.5 - 26.3]	14.6	[12.2 - 17.5]	0.48
**'CsPO38-4_unicycler.fasta'**	*Corynebacterium vitaeruminis* DSM 20294	13.5	[10.8 - 16.8]	23.8	[21.5 - 26.3]	13.9	[11.5 - 16.7]	11.11

Query	Subject	d_0	C.I. d_0	d_4	C.I. d_4	d_6	C.I. d_6	Diff. G+C Percent
**'CsPO104-5_unicycler.fasta'**	*Corynebacterium vitaeruminis* DSM 20294	13.5	[10.8 - 16.8]	23.8	[21.5 - 26.3]	13.9	[11.5 - 16.7]	11.11
**'CsPO105-5_unicycler.fasta'**	*Corynebacterium diphtheriae* subsp. *lausannense* CHUV2995	14.3	[11.5 - 17.7]	23.8	[21.5 - 26.2]	14.6	[12.2 - 17.5]	0.48
**'CsPO100-5_unicycler.fasta'**	*Corynebacterium vitaeruminis* DSM 20294	13.5	[10.8 - 16.8]	23.8	[21.5 - 26.3]	13.9	[11.5 - 16.7]	11.11
**'CsPO25-4_unicycler.fasta'**	*Corynebacterium diphtheriae* subsp. *lausannense* CHUV2995	14.3	[11.5 - 17.7]	23.8	[21.5 - 26.2]	14.6	[12.2 - 17.5]	0.48
**'CsPO39-4_unicycler.fasta'**	*Corynebacterium diphtheriae* subsp. *lausannense* CHUV2995	14.3	[11.5 - 17.7]	23.8	[21.5 - 26.2]	14.6	[12.2 - 17.5]	0.48
**'CsPO104-5_unicycler.fasta'**	*Corynebacterium diphtheriae* subsp. *lausannense* CHUV2995	14.3	[11.5 - 17.7]	23.8	[21.5 - 26.2]	14.6	[12.2 - 17.5]	0.48
**'CsPO38-4_unicycler.fasta'**	*Corynebacterium diphtheriae* subsp. *lausannense* CHUV2995	14.3	[11.5 - 17.7]	23.8	[21.5 - 26.2]	14.6	[12.2 - 17.5]	0.48
**'CsPO102-5_unicycler.fasta'**	*Corynebacterium vitaeruminis* DSM 20294	13.5	[10.8 - 16.8]	23.8	[21.5 - 26.3]	13.9	[11.5 - 16.7]	11.11
**'CsPO101-5_unicycler.fasta'**	*Corynebacterium diphtheriae* subsp. *lausannense* CHUV2995	14.3	[11.5 - 17.7]	23.8	[21.5 - 26.2]	14.6	[12.2 - 17.5]	0.48
**'CsPO101-5_unicycler.fasta'**	*Corynebacterium vitaeruminis* DSM 20294	13.5	[10.8 - 16.8]	23.8	[21.5 - 26.3]	13.9	[11.5 - 16.7]	11.11
**'Cs5182.fasta'**	*Corynebacterium diphtheriae* subsp. *lausannense* CHUV2995	14.4	[11.5 - 17.7]	23.7	[21.4 - 26.2]	14.6	[12.2 - 17.5]	0.5
**'CsPO102-5_unicycler.fasta'**	*Corynebacterium rouxii* FRC0190 T	14.3	[11.5 - 17.7]	23.3	[21.0 - 25.8]	14.6	[12.1 - 17.4]	1.19
**'CsPO100-5_unicycler.fasta'**	*Corynebacterium rouxii* FRC0190 T	14.3	[11.5 - 17.7]	23.2	[20.9 - 25.7]	14.6	[12.1 - 17.4]	1.19
**'CsPO38-4_unicycler.fasta'**	*Corynebacterium rouxii* FRC0190 T	14.3	[11.5 - 17.7]	23.2	[20.9 - 25.7]	14.6	[12.1 - 17.4]	1.19
**'Cs5182.fasta'**	*Corynebacterium rouxii* FRC0190 T	14.3	[11.5 - 17.7]	23.2	[20.9 - 25.7]	14.6	[12.1 - 17.4]	1.21
**'CsPO25-4_unicycler.fasta'**	*Corynebacterium rouxii* FRC0190 T	14.3	[11.5 - 17.7]	23.2	[20.9 - 25.7]	14.6	[12.1 - 17.4]	1.19
**'CsPO105-5_unicycler.fasta'**	*Corynebacterium rouxii* FRC0190 T	14.3	[11.5 - 17.7]	23.2	[20.9 - 25.7]	14.6	[12.1 - 17.4]	1.19
**'CsPO104-5_unicycler.fasta'**	*Corynebacterium rouxii* FRC0190 T	14.3	[11.5 - 17.7]	23.2	[20.9 - 25.7]	14.6	[12.1 - 17.4]	1.19
**'CsPO101-5_unicycler.fasta'**	*Corynebacterium rouxii* FRC0190 T	14.3	[11.5 - 17.7]	23.2	[20.9 - 25.7]	14.6	[12.1 - 17.4]	1.19
**'CsPO39-4_unicycler.fasta'**	*Corynebacterium rouxii* FRC0190 T	14.3	[11.5 - 17.7]	23.2	[20.9 - 25.7]	14.6	[12.1 - 17.4]	1.19
**'CsPO100-5_unicycler.fasta'**	*Corynebacterium diphtheriae* NCTC 11397	14.3	[11.4 - 17.7]	22.7	[20.4 - 25.2]	14.5	[12.1 - 17.4]	0.89
**'CsPO101-5_unicycler.fasta'**	*Corynebacterium diphtheriae* NCTC 11397	14.3	[11.4 - 17.7]	22.7	[20.4 - 25.2]	14.5	[12.1 - 17.4]	0.89
**'CsPO104-5_unicycler.fasta'**	*Corynebacterium diphtheriae* NCTC 11397	14.3	[11.4 - 17.7]	22.7	[20.4 - 25.2]	14.5	[12.1 - 17.4]	0.89
**'CsPO38-4_unicycler.fasta'**	*Corynebacterium diphtheriae* NCTC 11397	14.3	[11.4 - 17.7]	22.7	[20.4 - 25.2]	14.5	[12.1 - 17.4]	0.89
**'CsPO102-5_unicycler.fasta'**	*Corynebacterium belfantii* FRC0043	14.2	[11.4 - 17.6]	22.7	[20.4 - 25.2]	14.5	[12.0 - 17.3]	0.79

Query	Subject	d_0	C.I. d_0	d_4	C.I. d_4	d_6	C.I. d_6	Diff. G+C Percent
**'CsPO39-4_unicycler.fasta'**	*Corynebacterium diphtheriae* NCTC 11397	14.3	[11.4 - 17.7]	22.7	[20.4 - 25.2]	14.5	[12.1 - 17.4]	0.89
**'CsPO25-4_unicycler.fasta'**	*Corynebacterium diphtheriae* NCTC 11397	14.3	[11.4 - 17.7]	22.7	[20.4 - 25.1]	14.5	[12.1 - 17.4]	0.89
**'CsPO102-5_unicycler.fasta'**	*Corynebacterium diphtheriae* NCTC 11397	14.3	[11.4 - 17.7]	22.7	[20.4 - 25.2]	14.5	[12.1 - 17.4]	0.89
**'CsPO105-5_unicycler.fasta'**	*Corynebacterium diphtheriae* NCTC 11397	14.3	[11.4 - 17.7]	22.7	[20.4 - 25.2]	14.5	[12.1 - 17.4]	0.89
**'Cs5182.fasta'**	*Corynebacterium diphtheriae* NCTC 11397	14.3	[11.4 - 17.7]	22.7	[20.4 - 25.2]	14.5	[12.1 - 17.4]	0.91
**'CsPO101-5_unicycler.fasta'**	*Corynebacterium belfantii* FRC0043	14.2	[11.4 - 17.6]	22.6	[20.4 - 25.1]	14.5	[12.0 - 17.3]	0.79
**'CsPO100-5_unicycler.fasta'**	*Corynebacterium belfantii* FRC0043	14.2	[11.4 - 17.6]	22.6	[20.4 - 25.1]	14.5	[12.0 - 17.3]	0.79
**'CsPO39-4_unicycler.fasta'**	*Corynebacterium belfantii* FRC0043	14.2	[11.4 - 17.6]	22.6	[20.4 - 25.1]	14.5	[12.0 - 17.3]	0.79
**'CsPO105-5_unicycler.fasta'**	*Corynebacterium belfantii* FRC0043	14.2	[11.4 - 17.6]	22.6	[20.4 - 25.1]	14.5	[12.0 - 17.3]	0.79
**'CsPO25-4_unicycler.fasta'**	*Corynebacterium belfantii* FRC0043	14.2	[11.4 - 17.6]	22.6	[20.4 - 25.1]	14.5	[12.0 - 17.3]	0.79
**'CsPO104-5_unicycler.fasta'**	*Corynebacterium belfantii* FRC0043	14.2	[11.4 - 17.6]	22.6	[20.4 - 25.1]	14.5	[12.0 - 17.3]	0.79
**'CsPO38-4_unicycler.fasta'**	*Corynebacterium belfantii* FRC0043	14.2	[11.4 - 17.6]	22.6	[20.4 - 25.1]	14.5	[12.0 - 17.3]	0.79
**'Cs5182.fasta'**	*Corynebacterium belfantii* FRC0043	14.2	[11.4 - 17.6]	22.6	[20.4 - 25.1]	14.5	[12.0 - 17.3]	0.81
**'CsPO39-4_unicycler.fasta'**	*Corynebacterium diphtheriae* DSM 44123	14.2	[11.4 - 17.6]	22.1	[19.8 - 24.5]	14.5	[12.0 - 17.3]	0.88
**'CsPO102-5_unicycler.fasta'**	*Corynebacterium diphtheriae* DSM 44123	14.2	[11.4 - 17.6]	22.1	[19.8 - 24.5]	14.5	[12.0 - 17.3]	0.88
**'CsPO100-5_unicycler.fasta'**	*Corynebacterium diphtheriae* DSM 44123	14.2	[11.4 - 17.6]	22.1	[19.8 - 24.5]	14.5	[12.0 - 17.3]	0.88
**'CsPO105-5_unicycler.fasta'**	*Corynebacterium diphtheriae* DSM 44123	14.2	[11.4 - 17.6]	22.1	[19.8 - 24.5]	14.5	[12.0 - 17.3]	0.88
**'CsPO25-4_unicycler.fasta'**	*Corynebacterium diphtheriae* DSM 44123	14.2	[11.4 - 17.6]	22.1	[19.8 - 24.5]	14.5	[12.0 - 17.3]	0.88
**'Cs5182.fasta'**	*Corynebacterium diphtheriae* DSM 44123	14.2	[11.4 - 17.6]	22.1	[19.8 - 24.5]	14.5	[12.0 - 17.3]	0.9
**'CsPO101-5_unicycler.fasta'**	*Corynebacterium diphtheriae* DSM 44123	14.2	[11.4 - 17.6]	22.1	[19.8 - 24.5]	14.5	[12.0 - 17.3]	0.88
**'CsPO104-5_unicycler.fasta'**	*Corynebacterium diphtheriae* DSM 44123	14.2	[11.4 - 17.6]	22.1	[19.8 - 24.5]	14.5	[12.0 - 17.3]	0.88
**'CsPO38-4_unicycler.fasta'**	*Corynebacterium diphtheriae* DSM 44123	14.2	[11.4 - 17.6]	22.1	[19.8 - 24.5]	14.5	[12.0 - 17.3]	0.88

Table 4: Strains in your dataset

Joint dataset of automatically determined closest type strains (if this mode was chosen), manually selected type strains (if selected accordingly) and the provided user strains, if provided (marked in yellow).

Strain	Authority	Other deposits	Synonyms	Base pairs	Percent G+C	No. proteins	Goldstamp	Bioproject accession	Biosample accession	Assembly accession	IMG OID
Corynebacterium vitaeruminis DSM 20294	(Bechdel et al. 1928) Lanéelle et al. 1980	ATCC 10234; CCUG 28792; JCM 1323; IFO 12143; NBRC 12143; VKM B-1211; CIP 82.07; NCIB 9291; NCIMB 9291	*Brevibacterium vitaeruminis*; *Corynebacterium vitaeruminis*; *Flavobacterium vitarumen*	2931 780	65.5	2577	Gp0023683	PRJNA172966	SAMN03081455	GCA_000550805	2558860221
Corynebacterium diphtheriae subsp. *lausannense* CHUV2995	Tagini et al. 2019	CCUG 72509; DSM 107520	*Corynebacterium diphtheriae* subsp. *lausannense*	3060 363	53.9	3145	Gp0442955	PRJEB24256	SAMEA104679569	GCA_900312965	
Corynebacterium belfantii FRC0043	Dazas et al. 2018	DSM 105776; CIP 111412	*Corynebacterium belfanti*; *Corynebacterium belfantii*	2598 827	53.6	2557	Gp0364753	PRJEB22103	SAMEA104208677	GCA_900205605	
Corynebacterium silvaticum KL0182	Dangel et al. 2020	LMG 31313; DSM 109166; CIP 111672	*Corynebacterium silvaticum*	2548 487	54.4	2017		PRJNA517029	SAMN10039578	GCA_004382825	
Corynebacterium resistens DSM 45100	Otsuka et al. 2005 emend. Nouioui et al. 2018	CCUG 50093; JCM 12819; GTC 2026; SICGH 158	*Corynebacterium resistens*	2601 311	57.1	2171	Gp0003835	PRJNA39683	SAMN02603065	GCA_000177535	650716029
Corynebacterium diphtheriae DSM 44123	(Kruse 1886) Lehmann and Neumann 1896 emend. Nouioui et al. 2018	ATCC 27010; DSM 44123; NCTC 11397; CIP 100721	*Bacillus diphtheriae*; *Corynebacterium diphtheriae*; *Corynebacterium diphtheriae* subsp. *diphtheriae*	2364 574	53.5	2116		PRJNA296455	SAMN04099181	GCA_001913265	

Strain	Authority	Other deposits	Synonyms	Base pairs	Percent G+C	No. proteins	Goldstamp	Bioproject accession	Biosample accession	Assembly accession	IMG OID
Corynebacterium rouxii FRC0190 T	Badell et al. 2020	DSM 110354; CIP 111752	*Corynebacterium rouxii*	2451 019	53.2	2365		PRJNA224116	SAMEA5992727	GCF_902702935	
Corynebacterium pseudotuberculosis ATCC 19410	(Buchanan 1911) Eberson 1918 emend. Nouioui et al. 2018	ATCC 19410; CCUG 2806; DSM 20689; JCM 9389; IFO 15363; NBRC 15363; NCTC 3450; CIP 102968	*Bacillus pseudotuberculosis*; *Corynebacterium pseudotuberculosis*	2337 763	52.2	2146	Gp0223239	PRJNA382169	SAMN06701041	GCA_002155265	
Corynebacterium pseudotuberculosis DSM 20689	(Buchanan 1911) Eberson 1918 emend. Nouioui et al. 2018	ATCC 19410; CCUG 2806; DSM 20689; JCM 9389; IFO 15363; NBRC 15363; NCTC 3450; CIP 102968	*Bacillus pseudotuberculosis*; *Corynebacterium pseudotuberculosis*	2338 546	52.2	2084	Gp0220522	PRJNA442833	SAMN08778220	GCA_003634885	2756170169
Corynebacterium ulcerans NCTC 7910	(ex Gilbert and Stewart 1927) Riegel et al. 1995	ATCC 51799; CCUG 2708; DSM 46325; JCM 10387; CIP 106504	*Corynebacterium ulcerans*	2453 761	53.3	2178	Gp0262745	PRJEB6403	SAMEA4504038	GCA_900187135	
Corynebacterium kutscheri DSM 20755	(Migula 1900) Bergey et al. 1925 emend. Nouioui et al. 2018	ATCC 15677; CCUG 27535; DSM 20755; JCM 9385; IFO 15288; NBRC 15288; NCTC 11138; CIP 103423	*Bacterium kutscheri*; *Corynebacterium kutscheri*	2354 065	46.5	2047	Gp0110293	PRJNA276037	SAMN03365283	GCA_000980835	

Strain	Authority	Other deposits	Synonyms	Base pairs	Percent G+C	No. proteins	Goldstamp	Bioproject accession	Biosample accession	Assembly accession	IMG OID
Corynebacterium diphtheriae NCTC 11397	(Kruse 1886) Lehmann and Neumann 1896 emend. Nouioui et al. 2018	ATCC 27010; DSM 44123; NCTC 11397; CIP 100721	*Bacillus diphtheriae*; *Corynebacterium diphtheriae*; *Corynebacterium diphtheriae* subsp. *diphtheriae*	2463 666	53.5	2337	Gp0132011	PRJEB6403	SAMEA2517360	GCA_001457455	
Cs5182.fasta				2550 121	54.4	2520					
CsPO25-4_unicycler.fasta				2549 684	54.4	2526					
CsPO38-4_unicycler.fasta				2549 638	54.4	2530					
CsPO39-4_unicycler.fasta				2549 634	54.4	2533					
CsPO100-5_unicycler.fasta				2549 638	54.4	2533					
CsPO101-5_unicycler.fasta				2549 765	54.4	2529					
CsPO102-5_unicycler.fasta				2549 710	54.4	2528					
CsPO104-5_unicycler.fasta				2549 664	54.4	2529					
CsPO105-5_unicycler.fasta				2549 702	54.4	2530					

Methods, Results and References

The genome sequence data were uploaded to the Type (Strain) Genome Server (TYGS), a free bioinformatics platform available under https://tygs.dsmz.de, for a whole genome-based taxonomic analysis [1]. The results were provided by the TYGS on 2021-08-11. The TYGS analysis was subdivided into the following steps:

Determination of closely related type strains

Determination of closest type strain genomes was done in two complementary ways: First, all user genomes were compared against all type strain genomes available in the TYGS database via the MASH algorithm, a fast approximation of intergenomic relatedness [2], and, the ten type strains with the smallest MASH distances chosen per user genome. Second, an additional set of ten closely related type strains was determined via the 16S rDNA gene sequences. These were extracted from the user genomes using RNAmmer [3] and each sequence was subsequently BLASTed [4] against the 16S rDNA gene sequence of each of the currently 15065 type strains available in the TYGS database. This was used as a proxy to find the best 50 matching type strains (according to the bitscore) for each user genome and to subsequently calculate precise distances using the Genome BLAST Distance Phylogeny approach (GBDP) under the algorithm 'coverage' and distance formula d_5 [5]. These distances were finally used to determine the 10 closest type strain genomes for each of the user genomes.

Pairwise comparison of genome sequences

For the phylogenomic inference, all pairwise comparisons among the set of genomes were conducted using GBDP and accurate intergenomic distances inferred under the algorithm 'trimming' and distance formula d_5 [5]. 100 distance replicates were calculated each. Digital DDH values and confidence intervals were calculated using the recommended settings of the GGDC 2.1 [5].

Phylogenetic inference

The resulting intergenomic distances were used to infer a balanced minimum evolution tree with branch support via FASTME 2.1.6.1 including SPR postprocessing [6]. Branch support was inferred from 100 pseudo-bootstrap replicates each. The trees were rooted at the midpoint [7] and visualized with PhyD3 [8].

Type-based species and subspecies clustering

The type-based species clustering using a 70% dDDH radius around each of the 12 type strains was done as previously described [1]. The resulting groups are shown in Table 1 and 4. Subspecies clustering was done using a 79% dDDH threshold as previously introduced [9].

Results

Type-based species and subspecies clustering

The resulting species and subspecies clusters are listed in Table 4, whereas the taxonomic identification of the query strains is found in Table 1. Briefly, the clustering yielded 9 species clusters and the provided query strains were assigned to 1 of these. Moreover, user strains were located in 1 of 9 subspecies clusters.

Figure caption SSU tree

Figure 1. Tree inferred with FastME 2.1.6.1 [6] from GBDP distances calculated from 16S rDNA gene sequences. The branch lengths are scaled in terms of GBDP distance formula d_5. The numbers above branches are GBDP pseudo-bootstrap support values > 60 % from 100 replications, with an average branch support of 46.1 %. The tree was rooted at the midpoint [7].

Figure caption genome tree

Figure 2. Tree inferred with FastME 2.1.6.1 [6] from GBDP distances calculated from genome sequences. The branch lengths are scaled in terms of GBDP distance formula d_5. The numbers above branches are GBDP pseudo-bootstrap support values > 60 % from 100 replications, with an average branch support of 39.6 %. The tree was rooted at the midpoint [7].

References

[1] Meier-Kolthoff JP, Göker M. TYGS is an automated high-throughput platform for state-of-the-art genome-based taxonomy. Nat. Commun. 2019;10: 2182. DOI: 10.1038/s41467-019-10210-3

[2] Ondov BD, Treangen TJ, Melsted P, et al. Mash: Fast genome and metagenome distance estimation using MinHash. Genome Biol 2016;17: 1–14. DOI: 10.1186/s13059-016-0997-x

[3] Lagesen K, Hallin P. RNAmmer: consistent and rapid annotation of ribosomal RNA genes. Nucleic Acids Res. Oxford Univ Press; 2007;35: 3100–3108. DOI: 10.1093/nar/gkm160

[4] Camacho C, Coulouris G, Avagyan V, Ma N, Papadopoulos J, Bealer K, et al. BLAST+: architecture and applications. BMC Bioinformatics. 2009;10: 421. DOI: 10.1186/1471-2105-10-421

[5] Meier-Kolthoff JP, Auch AF, Klenk H-P, Göker M. Genome sequence-based species delimitation with confidence intervals and improved distance functions. BMC Bioinformatics. 2013;14: 60. DOI: 10.1186/1471-2105-14-60

[6] Lefort V, Desper R, Gascuel O. FastME 2.0: A comprehensive, accurate, and fast distance-based phylogeny inference program. Mol Biol Evol. 2015;32: 2798–2800. DOI: 10.1093/molbev/msv150

[7] Farris JS. Estimating phylogenetic trees from distance matrices. Am Nat. 1972;106: 645–667.

[8] Kreft L, Botzki A, Coppens F, Vandepoele K, Van Bel M. PhyD3: A phylogenetic tree viewer with extended phyloXML support for functional genomics data visualization. Bioinformatics. 2017;33: 2946–2947. DOI: 10.1093/bioinformatics/btx324

[9] Meier-Kolthoff JP, Hahnke RL, Petersen J, Scheuner C, Michael V, Fiebig A, et al. Complete genome sequence of DSM 30083^T, the type strain (U5/41^T) of *Escherichia coli*, and a proposal for delineating subspecies in microbial taxonomy. Stand Genomic Sci. 2014;9: 2. DOI: 10.1186/1944-3277-9-2
