# Supplementary figures and images for: Analysis of Corynebacterium silvaticum genomes from Portugal reveals a single cluster and a clade suggested to produce diphtheria toxin
Source: PeerJ. 2023 Mar 9;11:e14895. doi: 10.7717/peerj.14895 (PMC10008321; doi:10.7717/peerj.14895)

**A**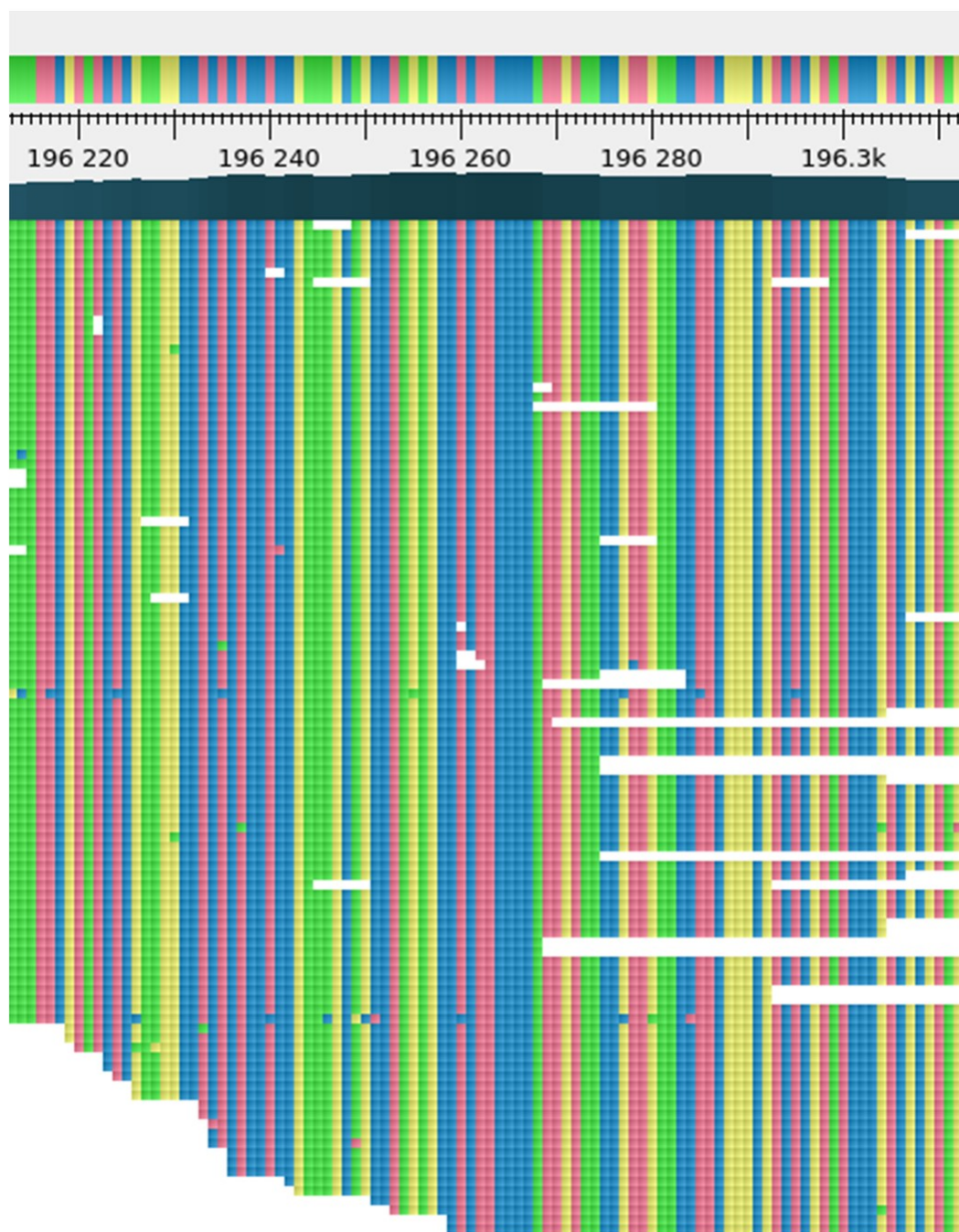**B**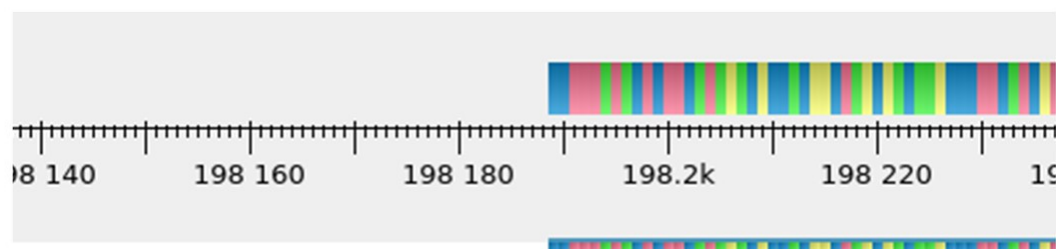

Supplement: Supplemental Information 14 [file peerj-11-14895-s014.pdf]
