# Supplementary material for: Analysis of Corynebacterium silvaticum genomes from Portugal reveals a single cluster and a clade suggested to produce diphtheria toxin
Source: PeerJ. 2023 Mar 9;11:e14895. doi: 10.7717/peerj.14895 (PMC10008321; doi:10.7717/peerj.14895)
Supplement: Supplemental Information 12 [file peerj-11-14895-s012.pdf]

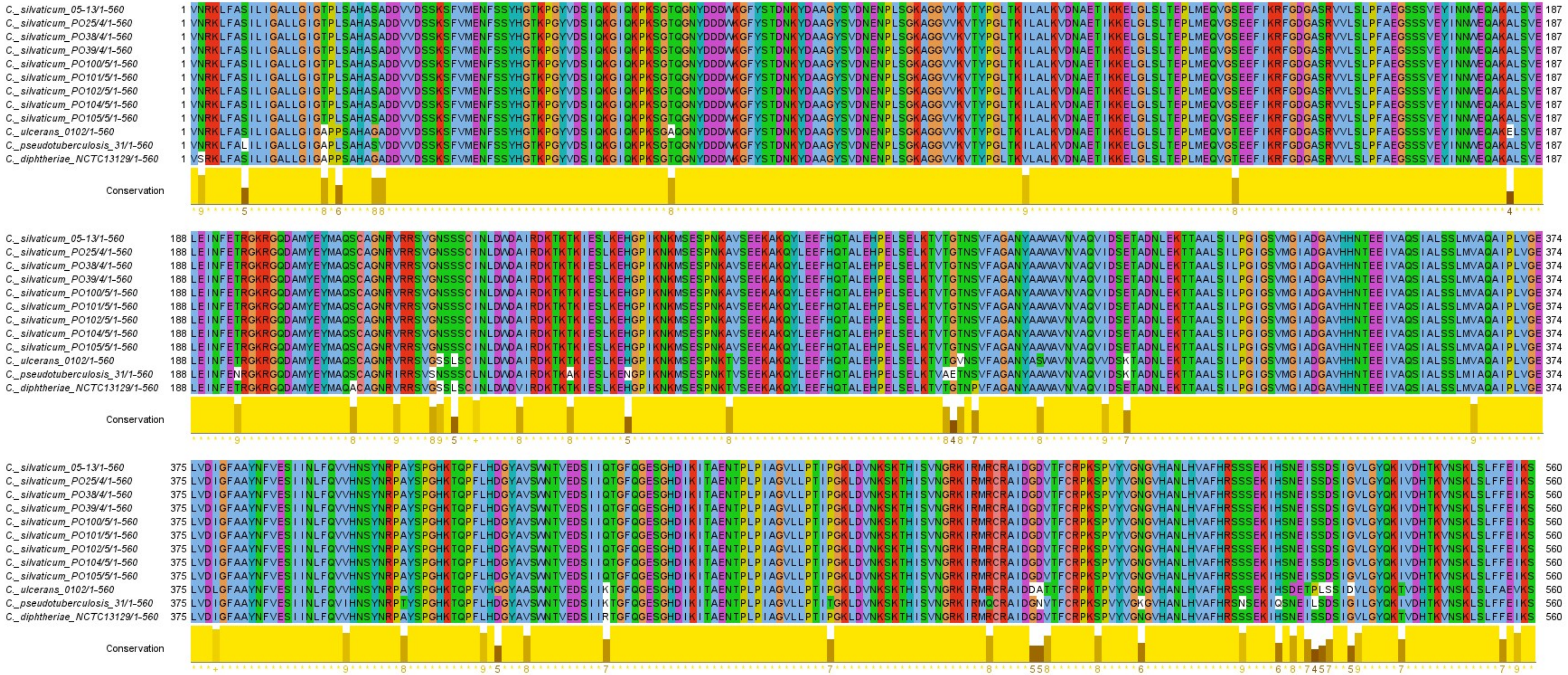
C._silvaticum_05-13/1-560
C._silvaticum_PO25/4/1-560
C._silvaticum_PO38/4/1-560
C._silvaticum_PO39/4/1-560
C._silvaticum_PO100/5/1-560
C._silvaticum_PO101/5/1-560
C._silvaticum_PO102/5/1-560
C._silvaticum_PO104/5/1-560
C._silvaticum_PO105/5/1-560
C._ulcerans_0102/1-560
C._pseudotuberculosis_31/1-560
C._diphtheriae_NCTC13129/1-560
Conservation
